# The microbial community structure and nitrogen cycle of high-altitude pristine saline lakes on the Qinghai-Tibetan plateau

**DOI:** 10.3389/fmicb.2024.1424368

**Published:** 2024-07-09

**Authors:** Zhe Zhao, Yuxiang Zhao, Federico Marotta, Maripat Xamxidin, Huan Li, Junquan Xu, Baolan Hu, Min Wu

**Affiliations:** ^1^College of Life Sciences, Zhejiang University, Hangzhou, China; ^2^Structural and Computational Biology Unit, European Molecular Biology Laboratory, Heidelberg, Germany; ^3^Environmental Microbiome Engineering and Biotechnology Laboratory, Center for Environmental Engineering Research, Department of Civil Engineering, The University of Hong Kong, Pokfulam, Hong Kong SAR, China; ^4^College of Environmental and Resource Sciences, Zhejiang University, Hangzhou, China; ^5^Lab of Plateau Ecology and Nature Conservation, The Altun Mountain National Nature Reserve, Xinjiang, China; ^6^Zhejiang Province Key Laboratory for Water Pollution Control and Environmental Safety, Hangzhou, China; ^7^Key Laboratory of Environment Remediation and Ecological Health, Ministry of Education, College of Environmental Resource Sciences, Zhejiang University, Hangzhou, China

**Keywords:** metagenomics, high-altitude pristine saline lakes, nitrogen cycle, Qinghai-Tibetan plateau, metagenome-assembled genomes

## Abstract

The nitrogen (N) cycle is the foundation of the biogeochemistry on Earth and plays a crucial role in global climate stability. It is one of the most important nutrient cycles in high-altitude lakes. The biogeochemistry of nitrogen is almost entirely dependent on redox reactions mediated by microorganisms. However, the nitrogen cycling of microbial communities in the high-altitude saline lakes of the Qinghai-Tibet Plateau (QTP), the world’s “third pole” has not been investigated extensively. In this study, we used a metagenomic approach to investigate the microbial communities in four high-altitude pristine saline lakes in the Altun mountain on the QTP. We observed that *Proteobacteria*, *Bacteroidota*, and *Actinobacteriota* were dominant in these lakes. We reconstructed 1,593 bacterial MAGs and 8 archaeal MAGs, 1,060 of which were found to contain nitrogen cycle related genes. Our analysis revealed that nitrite reduction, nitrogen fixation, and assimilatory nitrate reduction processes might be active in the lakes. Denitrification might be a major mechanism driving the potential nitrogen loss, while nitrification might be inactive. A wide variety of microorganisms in the lake, dominated by *Proteobacteria*, participate together in the nitrogen cycle. The prevalence of the dominant taxon *Yoonia* in these lakes may be attributed to its well-established nitrogen functions and the coupled proton dynamics. This study is the first to systematically investigate the structure and nitrogen function of the microbial community in the high-altitude pristine saline lakes in the Altun mountain on the QTP. As such, it contributes to a better comprehension of biogeochemistry of high-altitude saline lakes.

## 1 Introduction

Nitrogen is a vital element for organisms, critical for nucleic acid and protein synthesis, and is often the key element limiting the primary productivity of ecosystems (Falkowski, 1997). Nitrogen exists in the environment in many chemical compounds with oxidation states ranging from +5 to −3. The processes by which nitrogen converts between its different chemical forms are described in the nitrogen cycle, which is the foundation of the biogeochemistry on Earth (Martínez-Espinosa et al., 2011). The biogeochemistry of nitrogen is almost entirely dependent on redox reactions mediated by microorganisms (Canfield et al., 2010). The microorganisms are involved in seven main processes of nitrogen cycling in ecosystems: nitrogen fixation, ammonification, DNRA (dissimilatory nitrate reduction), assimilation, nitrification, denitrification, and anammox (Kuypers et al., 2018; Hutchins and Capone, 2022). The structure and function of microbial communities are closely related to the ecosystem function (Bottomley et al., 2012; Zhang et al., 2023).

The global nitrogen cycle has been severely disturbed since the 20th century by human activities including burning fossil fuels and applying artificial nitrogen fertilizers (Gruber and Galloway, 2008). Numerous environmental issues, including eutrophication of terrestrial and aquatic systems, an increase in the greenhouse gas nitrous oxide, and ozone depletion in the stratosphere, have been linked to the significant nitrogen input (Galloway et al., 2003; Lin and Lin, 2022). Over the past few decades, advancements in ecological studies have provided profound insights into the intricate pathways and mechanisms of nitrogen cycling in various terrestrial and aquatic environments, particularly in the soil and marine environment (Casciotti, 2016; Nelson et al., 2016; Tu et al., 2017; Pajares and Ramos, 2019). From unraveling the complexities of microbial nitrogen transformations to assessing the effects of human activities on nitrogen fluxes, recent research has significantly expanded our understanding of the complex networks of interactions among nitrogen-transforming microorganisms and their implications for the global biogeochemical nitrogen cycling. In the last 15 years, four new reactions have been discovered: oxidation of hydroxylamine to NO (Caranto and Lancaster, 2017), dismutation of NO to N_2_ and O_2_ (Ettwig et al., 2010), hydrazine synthesis (Kartal et al., 2011) and oxidation of hydrazine to N_2_ (Kartal et al., 2011). Numerous new metabolic processes were also discovered, including complete ammonia oxidation (comammox) to nitrate (Daims et al., 2015; Van Kessel et al., 2015) and phototrophic nitrite oxidation (Griffin et al., 2007). In addition, novel microorganisms, such as symbiotic heterotrophic nitrogen-fixing cyanobacteria (Thompson et al., 2012) and ammonia-oxidizing archaea (Konneke et al., 2005) were also identified. Nitrogen cycling in microbial communities is carried out by complex networks of microorganisms with great diversity. Studies of microbial communities in marine, agricultural and soil environments have found cyanobacteria (Gruber, 2008), *Nitrospina* spp. (Pachiadaki et al., 2017), “*Candidatus*. Methanoperedens” spp. (Vaksmaa et al., 2017) and other significant nitrogen-functioning microbial species. However, although nitrogen cycling in these environments has been studied in some depth, little research has been done on nitrogen cycling in high-altitude lakes, especially high-altitude saline lakes in the pristine areas of the QTP. Although some studies have analyzed the taxa composition, functions and distribution of microorganisms involved in specific nitrogen functions in lakes on the QTP (Jiang et al., 2009; Yang et al., 2012, 2013, 2015), the majority of the studies focused on a single functional taxon and lacked an understanding of the microbial community’s role in the overall nitrogen cycle. Furthermore, the investigation locations were situated in places with lower altitude and higher levels of human activity, rather than high-altitude pristine areas. It was not until 2023 that the first Shotgun metagenomics study for the microbial community of a lake above 4,000 meters above sea level (lake Bamucuo on the QTP) was reported (Wei et al., 2023). However, because this study only included data from one lake, it lacked systematization and did not comprehensively analyze the nitrogen cycling function of the lake’s microbial community. There is yet to be a comprehensive and systematic research of the microbial communities involved in nitrogen cycling in several lakes at an altitude of more than 4,000 m on the Altun Mountain of the Tibetan Plateau.

The “third pole,” the Qinghai-Tibetan Plateau (QTP), is the world’s highest plateau (average altitude of 4,500 m) and the largest plateau (2 × 10^6^ km^2^) (Yang et al., 2013). It contains thousands of lakes, which account for more than half of China’s total lake area (Kong et al., 2023). The plateau lake ecosystem is an important component of the plateau ecosystem. The QTP, along with the Arctic and Antarctic regions, is one of the most sensitive and vulnerable ecosystems in the world to global climate change, as well as a significant regulator of regional and global climate (Kuang and Jiao, 2016). However, despite being known as the “third pole,” there is far less data available for the QTP than for the Arctic and Antarctic (Qiu, 2008). Although many high-altitude lakes are located in areas of low human activity and are typically oligotrophic environments, in the context of a substantial increase in global nitrogen fluxes and an intensification of the greenhouse effect, and influenced by factors such as increased snow and ice melt, increased erosive power of rainfall, and atmospheric deposition, high-altitude lakes have continued to receive an increased amount of nitrogen from the environment (Wolfe et al., 2001; Vreca and Muri, 2006; Brahney et al., 2015; Zhang et al., 2019). On the QTP, data from the Lake Qinghai reveal that nitrogen from human activities enters the lake through atmospheric deposition, potentially impacting the aquatic ecosystem of the lake (Zhang et al., 2019). Four alpine lakes in the Sanjiangyuan Region (Lake Eling, Lake Longbao, Lake Sea Star, and Lake Zhaling) have shown an increasing tendency in numerous nitrogen and phosphorus indicators year after year (Lu et al., 2017). Over the last three decades, Lake Hurleg in the northeastern portion of the QTP has experienced an increase in nitrogen and phosphorus concentrations, although receiving very little direct human disturbance (Wu et al., 2021). Therefore, understanding the nitrogen cycling function of microbial communities in high-altitude lakes on the QTP in the context of increasing global nitrogen fluxes can contribute to our understanding of global biogeochemical cycling and microbial community responses to climate change.

In this context, the objectives of this study were (i) to investigate the structure of microbial communities in four high-altitude pristine saline lakes in the Altun Mountains on the QTP and (ii) to reveal the characteristics of the microbial nitrogen cycle and the microbial taxa involved in the nitrogen cycle in these lakes.

## 2 Materials and methods

### 2.1 Environmental samples collection

The study area is located in the Altun Shan National Nature Reserve on the northern edge of the QTP in China. From 2019 to 2021, we sampled four high-altitude pristine saline lakes in the Altun mountain (Lake Aqqikkol, Lake Ayakkum, Lake Jingyu and Lake Wusuxiao) multiple times (Supplementary Figures S1–S5). The sampling period covered the entire year, including April, July, October and November. Therefore, the samples were able to provide a comprehensive picture of the state of the lake microbial communities across time (Supplementary Table S1). 6–10 L of surface lake water were collected and promptly filtered using sterile filter membranes. The water was initially filtered with 10 μm membranes to remove bulk contaminants, then with 3 μm and 0.22 μm membranes, sequentially. The acquired filter membranes were folded inward twice into a 90-degree fan form, coated in sterile tin foil, and stored in plastic ziplock bags. The plastic ziplock bags were kept in dry ice and shipped to the company (Magigen, China) for DNA extraction and sequencing. Alkaline potassium persulfate digestion UV spectrophotometric method (HJ 636-2012) was used to determine the total nitrogen of the water samples (Huang et al., 2023). Environmental factors were measured using a YSI multiparameter digital water quality meter (YSI, America).

### 2.2 DNA extraction and metagenome sequencing

DNA from 30 lake water samples was extracted with the Advanced Water DNA Kit (ALFA-SEQ). Integrity and purity of extracted DNA were monitored on 1% agarose gels. PE150 strategy was performed on the Illumina HiSeq 2,500 high-throughput sequencing platform. The quality of raw sequencing data was evaluated by Illumina filters and Trimmomatic v0.36 (Bolger et al., 2014), which removed adapters, primers, and low-quality data (Zhao et al., 2023). The cleaned data were assessed for quality and basic information statistics with the SeqKit v0.9.3 (Shen et al., 2016).

### 2.3 Metagenomic assembly and binning

The cleaned data were *de novo* assembled using MEGAHIT v1.2.9 (Li et al., 2015) with the parameter --presets meta-sensitive (--min-count 1 --k-list 21,29,39,49,…,129,141). Contigs shorter than 2Kb were removed from the assembled metagenomic sequences. The assembled and filtered sequences were analyzed for quality and basic information statistics using the QUAST v5.0.2 (Gurevich et al., 2013). Metagenomic binning was performed using MetaBAT v2.12.1 (Kang et al., 2019) and CONCOCT v0.5.0 (Alneberg et al., 2014). The binning results produced by these two software programs were later integrated using DAS Tool v1.1.1 (Sieber et al., 2018) to build non-redundant MAGs (Metagenome-Assembled Genomes). The completeness and contamination of MAGs were assessed using CheckM v1.1.3 with “lineage_wf” workflow (Parks et al., 2015). For further analysis, only high- and medium-quality MAGs meeting the MIMAG standard (completeness ≥50% and contamination <10%) (Bowers et al., 2017) were retained. Taxonomic classification of MAGs was conducted using GTDB-Tk (v2.1.1, database r207) (Chaumeil et al., 2022). Because the samples were collected from special high-altitude pristine saline lake water habitats, the majority of MAGs could not be identified to the species level in the GTDB database. At the species level, ANI between MAGs was calculated using FastANI v1.32 (Jain et al., 2018) to distinguish between species with a 95% threshold (Richter and Rossello-Mora, 2009).

### 2.4 Data analysis

#### 2.4.1 Gene annotation and abundance calculation

Genes in assembled contigs or MAGs were predicted using Prodigal v2.6.3 (Hyatt et al., 2010) with the -p meta parameter. KAAS (KEGG automatic annotation server) (Moriya et al., 2007) was used to perform functional annotation of predicted genes using KEGG (Kyoto Encyclopedia of Genes and Genomes) database (Kanehisa and Goto, 2000). For the genomic features of a specific taxon, we used all the MAGs associated with that taxon. A gene was considered to be present in the taxon if it appeared in more than 25% of the MAGs or in high-quality MAGs with greater than 90% completeness (Nelson et al., 2020).

To evaluate the abundance of a given gene in the samples, we first performed a HMMER v3.3.1 (Eddy, 2011) search across contigs for both the gene of interest and the marker gene *rpsB* (ribosomal protein S2), assumed to be present in every bacterial genome. Then, we divided the total depth of the contigs containing the gene of interest by the total depth of the contigs containing the marker gene. This allowed us to interpret the gene abundance as the average copy number of that gene in the sample. Specifically, we calculated the gene abundance as:


$$\text{Gene}\;\text{Depth}=\frac{\sum_{q\in Q}{\text{RPKM}}_{q}}{\sum_{c\in C}{\text{RPKM}}_{c}}$$

where RPKM_i_ (Mortazavi et al., 2008) is the count of reads per kilobase million for contig i, Q is the set of contigs that contain the gene of interest, and C is the set of contigs that contain the marker gene.

#### 2.4.2 Genome-wide phylogenetic tree construction

Only MAGs with ≥70% genomic completeness and < 7% contamination were retained to construct the phylogenetic tree with the GTDB marker genes (120 bacterial marker genes or 122 archaeal marker genes). Sequence alignment was first performed using MAFFT v7.453 (Katoh and Standley, 2013), then again using MUSCLE v5.0.1278 (Edgar, 2004), and finally the aligned sequences were trimmed using trimAL v1.4 (Capella-Gutierrez et al., 2009). Maximum likelihood (ML) tree building was performed using RAxML v8.2.11 (Stamatakis, 2014) with the parameter -m PROTGAMMAAUTO -N autoMRE to automatically set the number of replications of tree building and to calculate the bootstraps. Phylogenetic trees were visualized using the iTOL (Interactive Tree Of Life) online tool (Letunic and Bork, 2007).

#### 2.4.3 MAGs abundance calculation

Reads from cleaned sequencing data for each sample were recruited to MAGs using the “mem” module of BWA v0.7.17 (Heng, 2013). The result files were formatted and sorted using SAMtools v1.7 (Danecek et al., 2021). The depth of each contig within each MAG for every sample, i.e., the count of reads mapped to each genomic locus, was calculated using the “jgi_summarise_bam_contig_depths” program contained in MetaBAT v2.12.1 (Kang et al., 2019). The aggregate depth of all contigs within a sample for each MAG, termed as “MAG Raw Depth,” was derived by summing the depths of individual contigs. This “MAG Raw Depth” was then normalized for the size of the MAG and the size of the sample sequencing reads data. The formula is as follows:


$$MAG\;\text{Depth}=\frac{\frac{MAG\;\text{Raw}\;\text{Depth}}{MAG\;\text{Size}}}{\text{Raw}\;\text{Reads}\;\text{Size}}$$

The resulting depth values can reflect the abundance of MAGs in the samples. Furthermore, these normalized results can be compared across different MAGs and samples.

#### 2.4.4 Species abundance in microbial communities

Species-level abundance was obtained using mOTUs v3 (Milanese et al., 2019) based on unassembled metagenomic sequencing data. This information was then utilized to assess the structural composition of the microbial communities in the samples.

#### 2.4.5 Statistical analysis

All statistical analyses were performed in the R programming language version 4.2 (R Core Team, 2013). The significance of the difference in the abundance of genes across samples was assessed using the wilcox.test() function. The plots were created using the ggplot2 package (Villanueva and Chen, 2019).

## 3 Results

### 3.1 The microbially mediated nitrogen cycle

**Figure 1:**
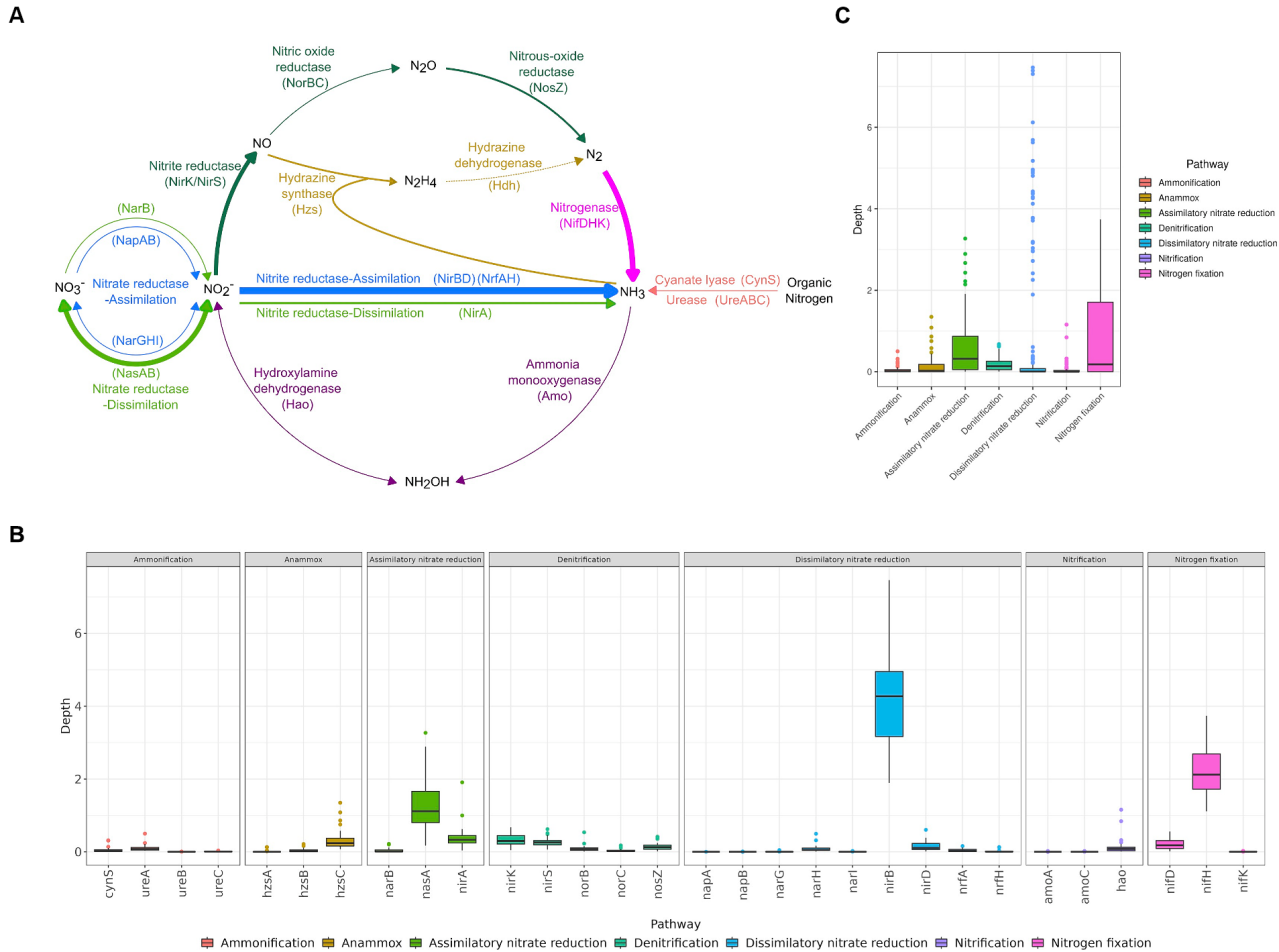
The abundance of functional genes involved in nitrogen cycling. **(A)** Nitrogen cycle, with the thickness of lines showing gene abundance and dashed lines showing that the genes were not found by annotation. **(B)** Abundance of nitrogen cycle related pathways. **(C)** Abundance of nitrogen cycle related genes.

The high-altitude pristine saline lakes in the Altun mountain are an oligonitrogenous environment, with a total nitrogen content of 0.2–0.5 mg/L. According to the metagenomic data, the *nirB*, *nifH*, and *nasA* genes were abundant in high-altitude pristine saline lakes in the Altun mountain, with median values of 4.27, 2.12, and 1.12, respectively (*p* < 0.05) (Figure 1; Supplementary Figure S6). Additionally, the *nirA*, *nirK*, and *nirS* genes also showed relatively high abundance, with median values of 0.33, 0.30, and 0.26, respectively (*p* < 0.05 compared to other genes, except for these three) (Figure 1; Supplementary Figure S6). Therefore, the nitrite reduction, nitrogen fixation, and assimilatory nitrate reduction processes might be active in the lakes. Especially the nitrite reduction process. The abundance of genes for the processes of dissimilatory nitrite reduction (*nirB*), assimilatory nitrite reduction (*nirA*) and nitrite reduction to nitric oxide (*nirK*/*nirS*) were all relatively high, implying the presence of numerous active nitrite reduction in the lake. Only the [FeMo]-nitrogenase gene (*nif*) was found in lake water samples; neither the [FeV]-nitrogenase gene (*vnf*) nor the [FeFe]-nitrogenase gene (*anf*) were discovered. In the four lakes, the depth profiles of nitrogen related genes showed similar patterns (Supplementary Figure S7). Among the six genes with high abundance, the *nirB*, *nifH*, *nirK*, and *nirS* genes showed no significant difference among the different lakes. The depth of the *nasA* gene was lower in Lake Ayakkum compared to Lake Aqqikkol and Lake Jingyu. Similarly, the depth of the *nirA* gene was lower in Lake Ayakkum than in Lake Aqqikkol (Supplementary Figure S8). This might suggest that dissimilatory nitrate reduction is relatively inactive in Lake Ayakkum compared to the other three lakes. In different sampling months (April, July, October, November), the *nirB*, *nifH*, *nirK*, and *nirS* genes also showed no significant difference. However, the depth of the *nasA* gene and the *nirA* gene was higher in October than in the other months (Supplementary Figure S9). These results indicated that the *nirB*, *nifH*, *nirK*, and *nirS* genes had a consistent and stable distribution in the lakes, whereas the *nasA* and *nirA* genes had a variable distribution across different lakes and varied in abundance over time.

### 3.2 Genome reconstruction and microbial community composition

**Figure 2:**
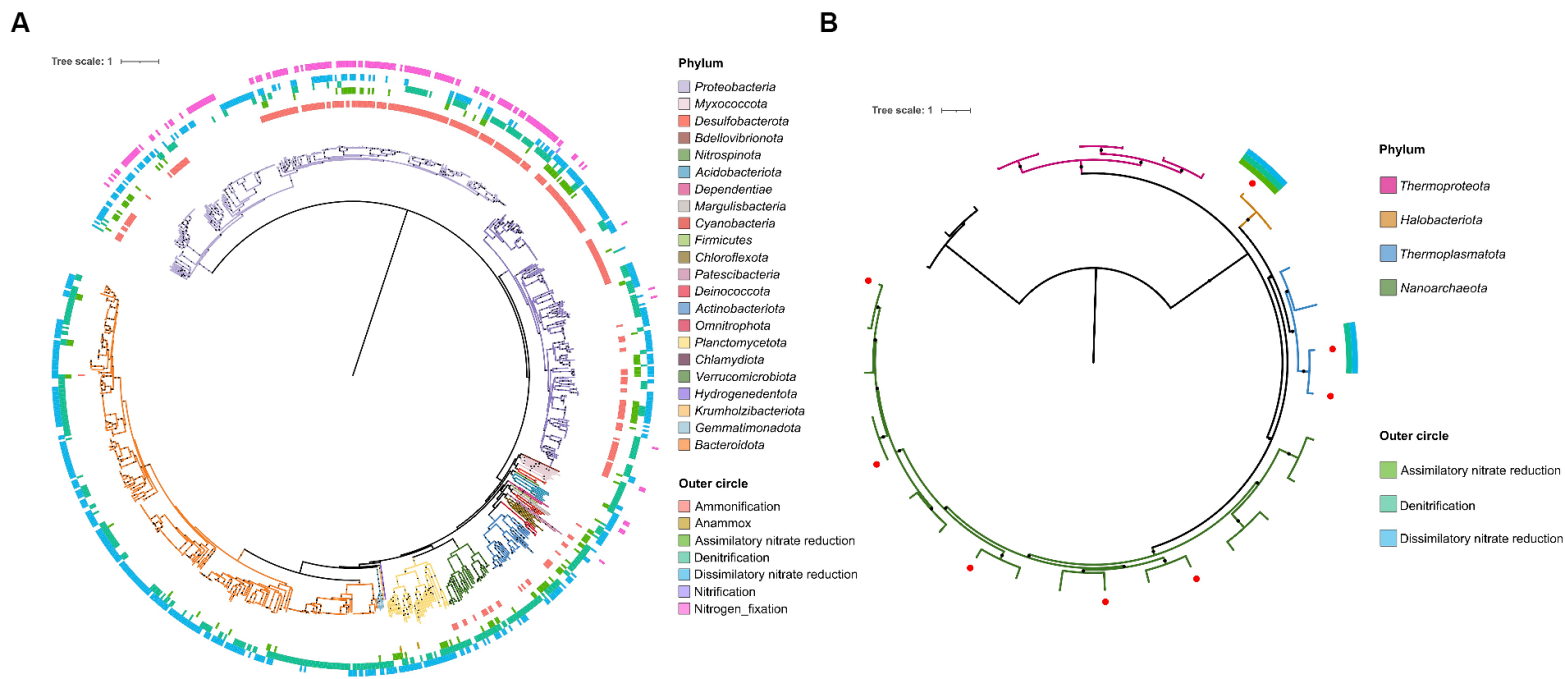
Phylogenetic trees of metagenome-assembled genomes (MAGs). **(A)** The phylogenetic tree of bacterial MAGs involved in the nitrogen cycle. **(B)** The phylogenetic tree of archaeal MAGs. Red dots indicate MAGs. The colors of the branches show different phyla. Solid circles on the branches represent bootstrap values ≥70% from 100 replicates. The outer circles show the nitrogen cycle related pathways encoded by each MAG, arranged from the center to the outside: Ammonification, Anammox, Assimilatory nitrate reduction, Denitrification, Dissimilatory nitrate reduction, Nitrification, and Nitrogen fixation.

A total of 1,975.8 GB of Illumina paired-end shotgun sequencing data was obtained from 30 lake water samples. The total size of *de novo* assembly (after removing contigs shorter than 2 KB) was 15.4 GB with average N50 7,618 bp (Supplementary Table S2). Binning the metagenomic data resulted in the reconstruction of 1,601 MAGs with quality higher than the medium quality standard (completeness ≥50% and contamination <10%) (Bowers et al., 2017). Among these MAGs, 1,593 were bacterial genomes and 8 were archaeal genomes (Figure 2; Supplementary Figure S10; Supplementary Table S3).

Of the 1,593 bacterial MAGs recovered from the lake water, a total of 24 phyla, 54 classes, 130 orders, 208 families, and 314 genera could be clearly categorized using the GTDB database. The ANI between MAGs was calculated, and a total of 701 species were obtained by dividing them with a 95% threshold. One MAG was classified as an unknown phylum, five MAGs were classified as unknown orders, 38 MAGs were classified as unknown families (2.4%), 376 MAGs were classified as unknown genera (23.6%), and 1,440 MAGs were classified as unknown species (90.4%). The 8 archaeal MAGs obtained from the lake water belonged to three phyla, three classes, three orders, and five families. They were all categorized as unknown species. Among them, two MAGs of the phylum *Thermoplasmatota* were classified as unknown families, and both were of good quality, suggesting the possibility of new taxonomic groups. The highest number of MAGs recovered from lake water samples was from *Proteobacteria* (696, including 377 *Alphaproteobacteria*, 318 *Gammaproteobacteria*, and 1 *Zetaproteobacteria*), followed by *Bacteroidota* (461) and *Actinobacteriota* (148) (Figure 2; Supplementary Figure S10; Supplementary Table S3). Based on the depth of the MAGs, the three most abundant phyla in the lakes were the same: *Bacteroidota* (33.1%), *Proteobacteria* (30.3%), and *Actinobacteriota* (18.8%) (Supplementary Figure S10; Supplementary Table S3). At the family level, *Rhodobacteraceae* (*Alphaproteobacteria*) had the most MAGs (201), followed by *Flavobacteriaceae* (*Bacteroidota*) (101), and *Microbacteriaceae* (*Actinobacteriota*) (81) (Supplementary Table S3). Based on the depth of the MAGs, the three most abundant families in the lakes were the same: *Flavobacteriaceae* (13.7%), *Microbacteriaceae* (13.5%), and *Rhodobacteraceae* (8.4%) (Supplementary Figure S10; Supplementary Table S3). In all four lakes, the highest number of MAGs was found in these three phyla. At the family level, the distribution of MAGs varied among the lakes (Table 1). Notably, *Flavobacteriaceae* and *Rhodobacteraceae* consistently ranked within the top five families in terms of MAG counts across all four lakes. Furthermore, *Balneolaceae*, *Burkholderiaceae*, *Cyclobacteriaceae*, *Microbacteriaceae*, and *Saprospiraceae* featured among the top five MAG-rich families in two lakes. All of the 14 genera present in all the four sampled lakes (Supplementary Table S4) encoded complete pathways related to low nitrogen in the two-component system (i.e., the NtrC family of nitrogen-regulation systems GlnL, GlnG, GlnA or NtrY, NtrX, NifA), whereas pathways associated with low phosphorus or low potassium were missing. Considering this, it could be hypothesized that functions related to nitrogen cycling may be one of the reasons why these taxa were able to prevail in these lakes. The classification results of MAGs suggested that microorganisms in high-altitude pristine saline lakes in the Altun mountain had a high species diversity, and a huge number of species had not yet been discovered and studied. Besides, nitrogen related functions played a significant role in the microbial communities of these lakes.

**Table 1:** The top 5 families in terms of counts of MAGs in the water of four lakes.

Lakes	Lake Aqqikkol (AQK)		Lake Ayakkum (AYK)		Lake Jingyu (JYH)		Lake Wusuxiao (WSX)	
No.	Family	Counts of MAGs	Family	Counts of MAGs	Family	Counts of MAGs	Family	Counts of MAGs
1	f__	17	*Rhodobacteraceae*	173	*Burkholderiaceae*	12	*Rhodobacteraceae*	12
2	*Planctomycetaceae*	8	*Flavobacteriaceae*	77	*Microbacteriaceae*	11	*Flavobacteriaceae*	8
3	*Cyclobacteriaceae*	8	*Microbacteriaceae*	63	*Flavobacteriaceae*	10	*Alteromonadaceae*	3
4	*Saprospiraceae*	7	*Saprospiraceae*	61	*Cyclobacteriaceae*	9	*Balneolaceae*	3
5	*Rhodobacteraceae*	7	*Burkholderiaceae*	55	*Rhodobacteraceae*	9	f__	3
6	*Flavobacteriaceae*	6	*Balneolaceae*	50	*Pseudohongiellaceae*	7	*Parvularculaceae*	3

### 3.3 MAGs contain nitrogen cycle related genes and their classification

A total of 1,060 MAGs in 24 phyla and 157 families were found to contain nitrogen cycle related genes (Figure 2; Supplementary Table S5). In all four lakes, the most abundant phyla, both in terms of number and depth of MAGs, were *Proteobacteria*, *Bacteroidota*, and *Actinobacteriota*. The six most abundant nitrogen circle related genes were also mainly encoded by these phyla. Specifically, the *nirB* gene was mainly encoded by *Bacteroidota* and *Proteobacteria*, the *nifH* gene by Proteobacteria, the *nasA* gene by Proteobacteria, the *nirA* gene by *Bacteroidota*, the *nirK* gene by *Proteobacteria*, *Bacteroidota*, and *Actinobacteriota*, and the *nirS* gene by *Proteobacteria* (Supplementary Table S5). The major phyla encoding the six most abundant nitrogen cycle related genes were broadly similar across each lake, though some differences were observed. In Lake Aqqikkol, the *nasA* gene was mainly encoded by the phyla *Proteobacteria*, *Bacteroidota*, and *Actinobacteriota*, while the *nirA* gene was mainly encoded by *Verrucomicrobiota*. In Lake Jingyu, the *nirA* gene was mainly encoded by *Bacteroidota* and *Proteobacteria*, and the MAG encoding the *nirS* gene was not annotated. In Lake Wusuxiao, the *nirA* gene was mainly encoded by *Cyanobacteria* (Supplementary Table S6). Besides, we found two archaeal MAGs that encoded nitrogen circle related genes (Figure 2; Supplementary Table S5). One belonged to *Halobacteriota* and encoded seven types of nitrogen circle related genes: *napA*, *narG*, *narH*, *nirA*, *nirK*, *norB*, and *nosZ*. The other one belonged to *Thermoplasmatota* and encoded *napA* and *nirK* genes. (Figure 2; Supplementary Table S5).

**Figure 3:**
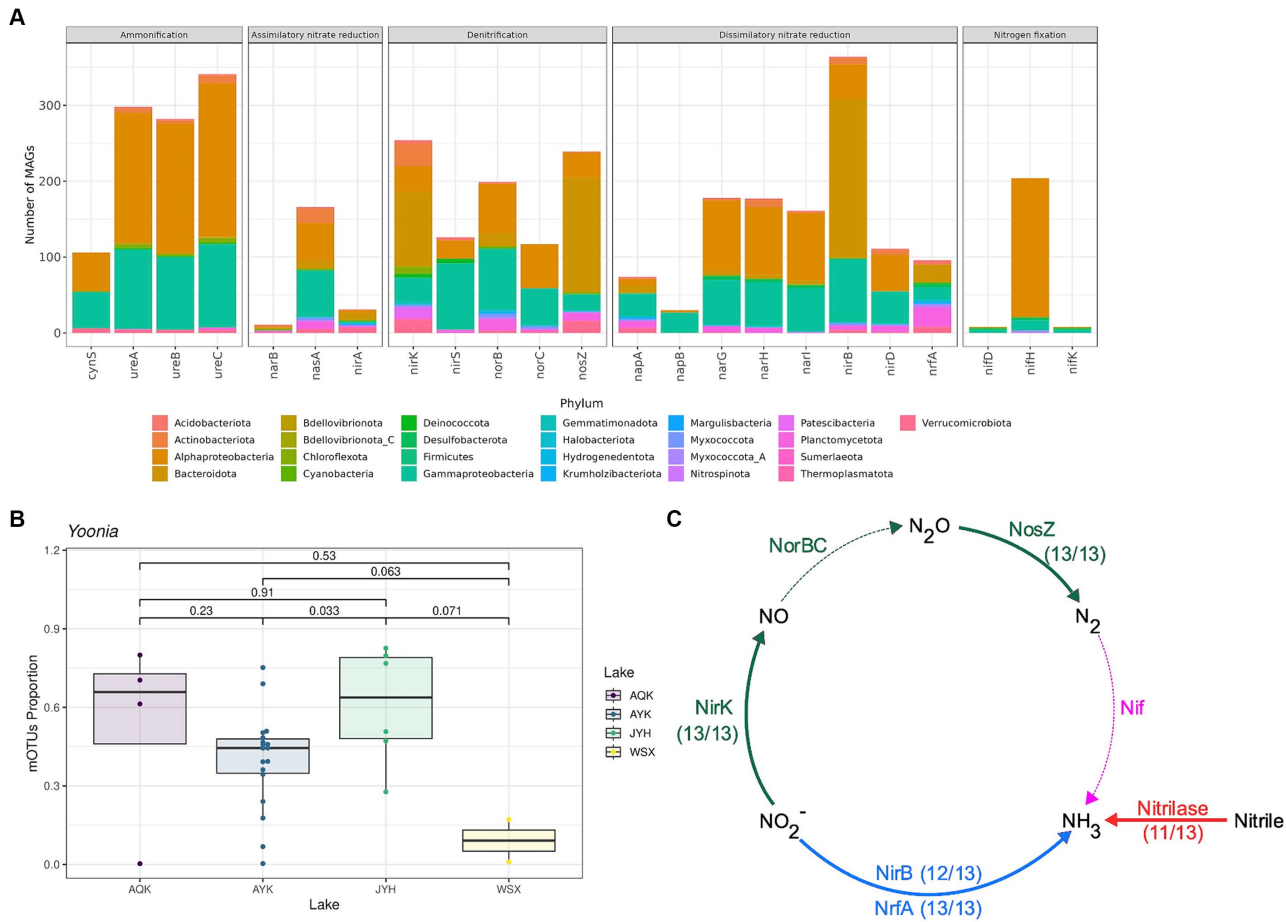
Species encoding genes with nitrogen cycle functions. **(A)** Number of MAGs encoding genes with nitrogen cycle functions in each phylum. **(B)** Abundance of *Yoonia* (*Alphaproteobacteria*) encoding nitrogenase in four high-altitude pristine lakes. **(C)** Number of MAGs encoding *nirB*, *nosZ*, and *nirK* genes in the total of 13 MAGs of *Cyclobacterium* (*Bacteroidota*). The dashed lines show that the genes were not found by annotation.

**Figure 4:**
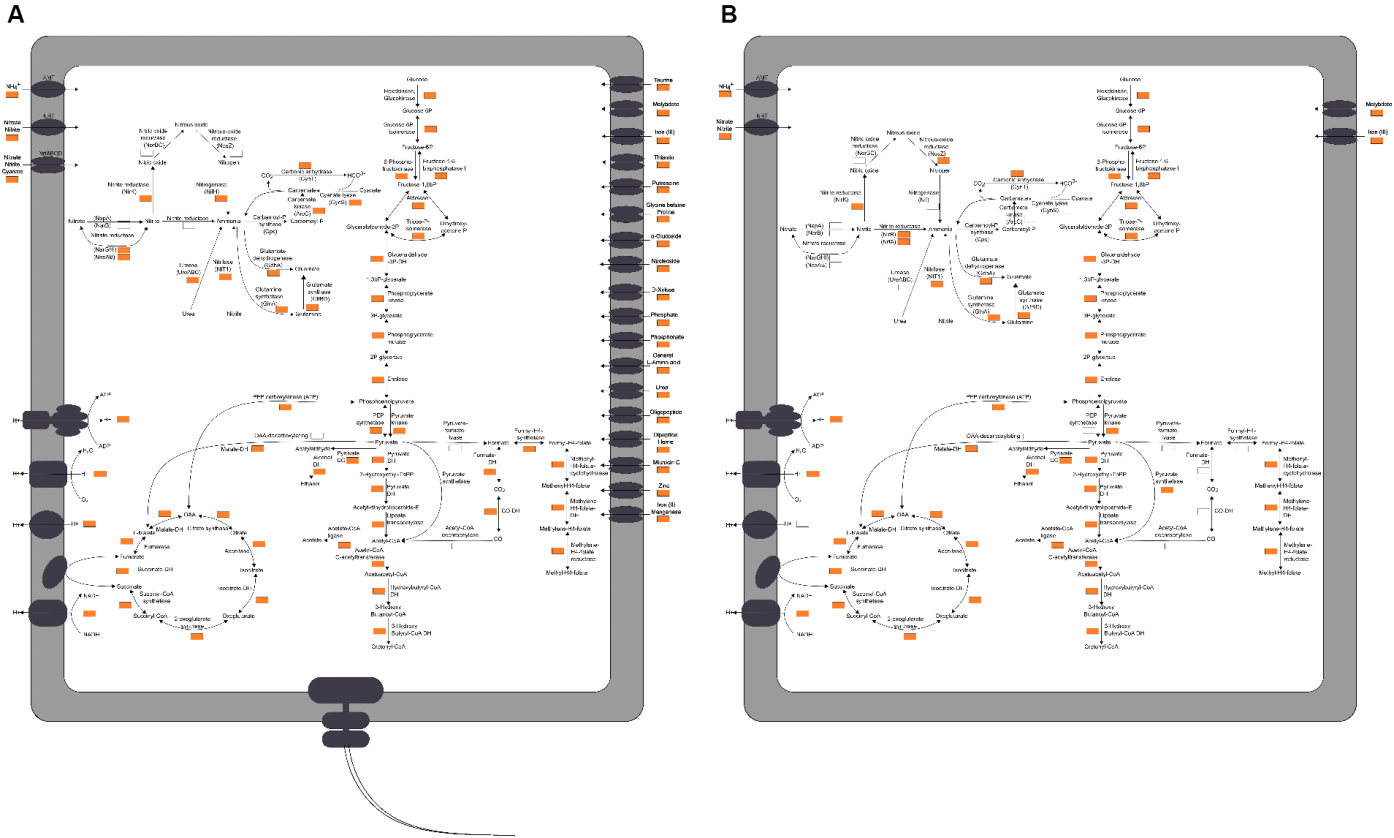
The proposed model of nitrogen metabolism in *Yoonia* and *Cyclobacterium*. Nitrogen cycle and coupled carbon cycle, oxidative phosphorylation and transmembrane transporters. A gene was considered to be present in the taxon if it appeared in more than 25% of the MAGs or in high-quality MAGs with greater than 90% completeness. **(A)** *Yoonia*. **(B)** *Cyclobacterium*.

The genus *Yoonia* of *Alphaproteobacteria*, *Rhodobacterales*, *Rhodobacteraceae* was the most prevalent taxon in the majority of lake samples, accounting for 17 to 80% of the microbial community (Figure 3B). This genus was more abundant in Lake Aqqikkol and Lake Jingyu, and less abundant in Lake Ayakkum and Lake Wusuxiao (Figure 3B). *Yoonia* encoded a variety of nitrogen cycle functional genes, including the [FeMo]-nitrogenase *nifH* gene, the *nirK* gene, the *narGHI* genes, and the *nasAB* genes, etc. (Figure 4A). It contributed an average of 23.9% of the depth of the *nirH* gene, 47.2% of the depth of the *nasA* gene, and 23.9% of the depth of the *nirA* gene in the microbial communities of the lakes (Supplementary Figure S11). Therefore, *Yoonia* might play an important role in the nitrogen cycle of high-altitude pristine saline lakes. *Bacteroidota* mainly encoded *nirB*, *nosZ*, and *nirK* genes in the lake water (Figure 3A), and *Cyclobacterium* (*Bacteroidota*, *Bacteroidia*, *Cytophagales*, *Cyclobacteriacea*) was a typical genus of *Bacteroidota* that encoded all these three genes simultaneously (Figure 3C).

### 3.4 Nitrogen cycle related genes in *Yoonia* and *Cyclobacterium*

*Yoonia* was the genus with the greatest abundance in lake water samples. There were five mOTUs within the genus *Yoonia*, including four species. In lake water samples, two mOTUs of *Y. vestfoldensis*, [ref_mOTU_v3_10885] and [ref_mOTU_v3_06935], accounted for 99% of the overall abundance of *Yoonia*. *Y. vestfoldensis* strains were recovered from microbial mats in Antarctic lakes, indicating its preference for pristine habitats (Van Trappen et al., 2004). It has been reported that *Yoonia* dominates the microbial community in the polar ocean with a proportion of approximately 11.1% (Zeng et al., 2022; Ding et al., 2023). However, the strong dominance (17–80%) of *Yoonia* in the lakes of the Altun mountain has not been observed in any other environment, thus making *Yoonia* a unique geospecific taxon in this setting. A total of 27 MAGs of *Yoonia* were recovered. Of these 27 MAGs, 11 were classified as invalid species name *Y*. sp018402175 and the remainders could not be categorized at the species level in the GTDB database.

*Yoonia* had the ability to convert inorganic nitrogen into amino acids. In addition, it encoded cyanase (CynS), urease (UreABC) and nitrilase (NIT1), which could use cyanate, urea and nitrile as nitrogen sources. This might favor its survival in low-nitrogen environments. As regards the nitrogen transport system, *Yoonia* encoded the Amt family ammonium transporter (AMT), the NNP family nitrate/nitrite transporter (NRT) and the nitrate/nitrite/Cyanate ABC transport system (NrtABCD). *Yoonia* had the ability to efficiently take up nitrogen from its surroundings (Figure 4A).

*Yoonia* encoded membrane-bound respiratory nitrate reductases (NarGHI), which is a membrane protein complex consisting of three different subunits. The NarI subunit anchors the NarG and NarH subunits, which are situated in the cytoplasm, to the membrane. NarG and NarH form the reductase module, receiving electrons from NarI, an integral membrane protein that binds two *b*-type hemes and draws electrons from the menaquinol pool to NarH. Menaquinol is oxidized at the periplasmic side of NarI, where electrons are transferred from the haem on the P side to the haem on the N side, ultimately reducing nitrate at the N side of the cytoplasmic membrane. The electrogenic (or proton) motive of NarGHI is caused by the transmembrane charge separation. The nitrate reduction by NarGHI is coupled to electron input from formate via the nitrate-inducible formate dehydrogenase (Fdh-N), forming the full proton motive redox loop (Simon et al., 2008).

*Yoonia* encoded the [FeMo]-nitrogenase (NifH), which uses eight electrons and 16 ATP to reduce each N_2_ molecule to two NH_3_ molecules (Sohm et al., 2011). Electrons and ATP can be obtained via coupling with oxidative phosphorylation. However, a greater challenge for nitrogen fixation is the management of oxygen. Since nitrogenase is oxygen-sensitive, aerobic nitrogen-fixing bacteria must employ techniques to protect nitrogenase from oxygen (Inomura et al., 2017). *Yoonia* is exposed to lower oxygen pressures because high altitudes are low-oxygen habitats. *Yoonia* encoded Fe-Mn family superoxide dismutase (SOD2), which may help protect its nitrogenase. Additionally, it has been found that aquatic heterotrophic organisms can fix nitrogen as long as there is a sufficient amount of carbohydrate substrate (Bentzon-Tilia et al., 2015). *Yoonia*’s ability to use a wide variety of carbon substrates, as well as its extensive transport systems, might also potentially be helpful for its nitrogen-fixing function.

*Yoonia* encoded not only a protein system associated with bacterial chemotaxis, namely: methyl-accepting chemotaxis protein (MCP), chemotaxis protein (Che), flagellar motor switch protein (Fli), and chemotaxis protein (Mot), but also four transmembrane chemoreceptors homologous to *Escherichia coli*, namely: serine sensor receptor (Tsr), aspartate sensor receptor (Tar), ribose and galactose sensor (Trg), and peptide sensor receptor (Tap) (Lane et al., 2006). *Yoonia* also encoded genes for flagellar assembly, therefore it might be motile. *Yoonia* might be more capable of sensing chemicals and chemotaxis, allowing it have a greater ability to thrive in the oligonitrogenous lake environment.

A total of 13 MAGs of *Cyclobacterium* were recovered. Using the GTDB database, all of these MAGs could not be categorized at the species level. Except for organic nitrogen synthase like GlnA, GltBD and GdhA and nitrogen-containing compound hydrolase NIT1, as a typical genus of *Bacteroidota*, *Cyclobacterium* also encoded *nirB*, *nrfA*, *nosZ*, and *nirK* genes (Figure 4).

The soluble cytoplasmic NADH-dependent nitrite reductase (Nir) catalyzes the reduction of nitrite to ammonium (Macdonald et al., 1985). Nir does not generate a proton gradient, but instead specifically uses NADH as the electron donor. NirB is the catalytic subunit. Cytochrome *c*-552 nitrite reductase (Nrf) is a soluble periplasmic enzyme. Different species may use NrfH protein or a complex consisting of NrfB, NrfC and NrfD proteins as the electron donor for NrfA. These are two genetically distinct systems, but both use NrfA as the catalytic enzyme (Lockwood et al., 2011). Both Nir and Nrf catalyze dissimilatory nitrite reduction to ammonium, which is the critical reaction in the dissimilatory nitrate reduction to ammonium (DNRA). *Cyclobacterium* may grow by coupling DNRA with the oxidation of electron donors such as organic substances and sulfide (Brunet and Garcia-Gil, 1996). Encoding multiple kinds of nitrite reductases may provide strains with enhanced environmental adaptation. *Escherichia coli* encodes both Nir and Nrf. Their expression is complementary: in environments with low nitrate or nitrite concentrations, *E. coli* synthesizes Nrf; in environments with high nitrate or nitrite concentrations, *E. coli* synthesizes Nir; and in environments with moderate nitrate concentrations, both enzymes are produced (Wang and Gunsalus, 2000). The coding and expression of these enzymes gives *E. coli* the ability to cope with a wide range of nitrate and nitrite concentrations in various settings. A similar pattern may exist in *Cyclobacterium*. NirK is located in the periplasm and use a *c*-type cytochrome as its electron source (Helen et al., 2016). NosZ is also located in the periplasm and use a *c*-type cytochrome or accessory proteins as its electron source (Wunsch and Zumft, 2005). Neither of them directly contributes to energy conservation through the proton motive force.

*Yoonia* encoded nitrate reduction but not nitrite reduction, and *Cyclobacterium* did not encode nitrate reduction but does encoded nitrite reduction. *Cyclobacterium* also encoded *nosZ* which was not encoded by *Yoonia*. *Yoonia* and *Cyclobacterium* is a good example to show that the classical process of nitrogen transformation is seldom accomplished by one single microorganism. In microbial communities, the nitrogen cycling is usually engaged by a wide variety of microorganisms.

## 4 Discussion

Nitrogenase (Nif) genes was found in all four lakes, with a total of 204 MAGs encoding nitrogenase genes belonging to four phyla: *Proteobacteria*, *Cyanobacteria*, *Myxococcota* and *Desulfobacterota* (Supplementary Table S5). *Proteobacteria* accounted for the majority, with 197 MAGs that encoded nitrogenase being extensively dispersed among the four lakes. High abundance of the *nifH* gene was observed in high-altitude pristine saline lakes in the Altun Mountains on the QTP, implying active nitrogen fixation. These lakes are oligonitrogenous environments. In such environments, microbial nitrogen fixation is an important source of nitrogen input to the ecosystem (Church et al., 2005). High diversity of the *nifH* gene has been observed in oligonitrogenous environments like Antarctica and the Arctic (Jungblut and Neilan, 2010; Díez et al., 2012). Additionally, the *nifH* gene of *Alphaproteobacteria* has been detected in the oligotrophic waters of the Atlantic and Pacific Oceans and alpine lake lakes (Zehr et al., 1998; Wang et al., 2020; Li et al., 2024). This is consistent with our findings that *Alphaproteobacteria* might assume a nitrogen-fixing function in oligonitrogenous aquatic environments. *Rhodobacteraceae* (*Yoonia*’s family, *Alphaproteobacteria*) was the only family with nitrogenase (Nif) genes annotated in all four lakes. Furthermore, *Rhodobacteraceae* was one of the most abundant taxa in the four lakes. Therefore, it is reasonable to hypothesize that *Rhodobacteraceae* is the main bearer of the nitrogen fixation function that is prevalent in the high-altitude pristine saline lakes in the Altun mountain. In addition to this family, each lake has its own specific nitrogen-fixing taxa.

In the denitrification pathway, nitrite reductase (NirK/NirS) and nitrous oxide reductase (NosZ) genes were more abundant, but nitric oxide reductase (NorBC) genes were less abundant (Figure 1). Similarly, a total of 115 MAGs encoding both subunits of nitric oxide reductase (NorBC) belonging to 6 phyla and 24 families were found, which was lower in number and variety compared to the taxa encoding nitrite reductase (NirK/NirS) (310 MAGs belonging to 17 phyla and 77 families) and nitrous oxide reductase (NosZ) (239 MAGs, belonging to 10 phyla and 10 families) (Supplementary Table S5). The low abundance and limited distribution of nitric oxide reductase genes (*norBC*) implied that the microbial community was weak in reducing nitrous oxide to nitrous oxide, whereas the high abundance and wide distribution of nitrous oxide reductase gene (*nosZ*) implied that the microbial community possessed a relatively high potential for reducing nitrous oxide. Therefore, the microbial community in high-altitude pristine saline lakes in the Altun mountain may prefer to release the end product nitrogen gas after completing the reduction of nitrous oxide in the process of denitrification.

The hydrazine synthase gene (*hzs*) was found to be less abundant in the lake than the nitrite reductase genes (*nirK*/*nirS*) (Figure 1; Supplementary Figure S12), and only four MAGs of *Planctomycetota* were found to encode the *hzs* gene (Supplementary Table S5). Therefore, anammox may not be active in the lakes, however denitrification may be a major mechanism driving the potential nitrogen loss in the high-altitude pristine saline lakes in the Altun mountain, which is consistent with the findings from high-altitude rivers on the QTP (Zhang et al., 2021). The abundance of the *nirK* gene in the lake water was similar to that of the *nirS* gene (Figure 1; Supplementary Figure S12), indicating that the denitrifier communities in the lake water were the mixed *nirK*/*nirS* type. Usually, *nirK*- and *nirS*-type denitrifier communities have different species compositions and respond differently to different environmental factors, which leads to changes in the species structure of the communities, and consequently affects the nitrogen loss process in the environment (Chen et al., 2021; Wang et al., 2021). The vast majority of MAGs encoding the nitrite reductase gene (*nirK*/*nirS*) (303 MAGs, 97.7%) encoded only one type of nitrite reductase gene, with only a few MAGs encoding both *nirK* and *nirS* genes (7 MAGs, 2.3%, 4 *Gammaproteobacteria*, 2 *Deinococcota*, and 1 *Acidobacteriae*). Recent research has found that the two types of *nir* genes are not mutually exclusive (Graf et al., 2014), however the functions of two different nitrite reductase types in one organism still need to be confirmed by further studies (Helen et al., 2016). Interestingly, 27 MAGs encoding *nirS*/*nirK* genes were found in *Rhodobacteraceae*, of which 23 (belonging to four genera *Yoonia*, *Roseo*var*ius*, *Flavimaricola* and *Cypionkella*) also encoded the nitrogenase gene (*nif*). These strains had the potential to both increase and decrease nitrogen in the environment.

Microbial communities in the high-altitude pristine saline lakes in the Altun mountain encoded all three types of nitrate reductase common in prokaryotes. A total of 341 MAGs encoding at least one type of nitrate reductase were annotated from all lake water samples, accounting for 21.3% of the total number of MAGs (1,601) and belonging to 17 phyla and 88 families. The majority of them encoded only one type of nitrate reductase, and there were 47 MAGs (13.78%) with more than one kind of nitrate reductase. The encoding of more than one types of nitrate reductase was observed mainly in *Proteobacteria*, especially in *Gammaproteobacteria*. Species encoding each nitrate reductase had considerable diversity, with significant variations in species composition among lakes. This implied that, whereas nitrate reduction to nitrite was a prevalent pathway in the lakes, different microbial taxa might achieve it in different lakes. It is important to note that the boundary between assimilatory and dissimilatory nitrate reduction is not absolute. Nitrite produced by assimilatory nitrate reduction can be further reduced in the respiratory chain. Experiments have demonstrated that *Mycobacterium tuberculosis* can use the NarGHI complex for assimilatory nitrate reduction (Malm et al., 2009).

Notably, although nitrate reductase (NarGHI) catalyzes nitrate reduction to nitrite, and nitrite oxidoreductase (NxrAB) catalyzes nitrite oxidation to nitrate, *narGHI* and *nxrAB* genes are homologs (Simon and Klotz, 2013). In the KEGG database, *narG* and *nxrA* genes share the same KO ID, as do *narH* and *nxrB* genes. As a result, they are difficult to distinguish from each other through annotations. Therefore, we did not single out the *nxr* genes, and the results annotated to each of these KOs were counted as *narGHI* genes.

Microbial communities in the high-altitude pristine saline lakes in the Altun mountain also encoded all three types of nitrite reductase common in prokaryotes. A total of 206 MAGs encoding one or more nitrite reductase genes were annotated from all lake water samples, accounting for 12.87% of the total number of MAGs (1,601) and belonging to 18 phyla and 61 families. The majority of them encoded only one type of nitrite reductase, and there were only18 MAGs (8.74%) with more than one kind of nitrite reductase, including *Cyclobacterium*, which encoded two kinds. Similar to the microbial taxa encoding nitrate reductase, species encoding each nitrite reductase had considerable diversity, with significant variations in species composition among lakes. Although encoding multiple types of nitrite reductases may provide strains with adaptations to cope with the wide range of nitrate and nitrite concentrations in the environment (Wang and Gunsalus, 2000), it may be that because nitrate and nitrite concentrations in high-altitude pristine saline lakes remain stable and low for long periods of time, microorganisms living in the water do not have the need to cope with drastic changes in nitrate and nitrite concentrations, and therefore do not require the complementary action of multiple enzymes.

In all lake water samples, a total of 424 MAGs were annotated with the potential to participate in the process of assimilatory or dissimilatory nitrate reduction, i.e., encoding nitrate reductase and/or nitrite reductase. There were 123 MAGs encoding both nitrate lyase and nitrite reductase, 218 MAGs encoding only nitrate reductase, and 83 MAGs encoding only nitrite reductase. Even taking into account missing genes due to incomplete MAGs, there are still many microorganisms that in fact encode only one functional enzyme and complete only one step of the catalytic reaction in the lakes. Because of the metabolic diversity of microorganisms and the complicated network of interactions in their communities, the classical process of nitrogen transformation is seldom accomplished by one single microorganism. Microbial communities are the units that carry out the nitrogen cycle in the environment, and communities are able to recycle nitrogen more efficiently than individual microbes. In microbial communities, the wide variety of microorganisms involved in nitrogen transformation processes generate complicated nitrogen cycling networks. To achieve optimal growth, each microbe has unique physiological demands and performs distinct nitrogen cycling functions (Kuypers et al., 2018).

The number of MAGs encoding the *napA* gene was detected to be only half of the number of MAGs encoding the *narGHI* genes, but the number of families to which they belonged was similar to the number of families to which MAGs encoding the *narGHI* genes belonged, and the number of phyla to which they belonged was even greater than that of the phyla to which MAGs encoding the *narGHI* genes belonged. It suggested that the distribution pattern of microorganisms encoding the *napA* gene in high-altitude pristine saline lakes in the Altun mountain was dispersed at the family level and above, with the *napA* gene encoded in microorganisms of many families but only a few strains of microorganisms in each family rather than universally. This distribution pattern could suggest the potential for the transfer of the *napA* gene across high-level taxonomic units, or gene acquisition and loss events that occurred relatively early in the evolutionary process. The abundance of the *amo* gene in the lake water was low and only one MAG encoding *amo* gene was found, belonging to *Nitrosomonas* in *Nitrosomonadaceae* of *Gammaproteobacteria*. It was implied that nitrification was not abundant in the lake water microbial community of high-altitude pristine saline lakes in the Altun mountain, although it might exist.

We noticed that for some genes, such as *nirB*, *nifH*, and *nasA*, the depth of these genes and the number and depth of the MAGs encoding these genes were consistent. However, for urease (*ureABC* genes), the gene depth was low, yet we found many MAGs encoding these genes (Figures 1, 3A). This might be due to binning biases. While powerful, the binning process in metagenomics is not without its biases. Nelson et al. (2020) found that repeat sequences and regions with variant nucleotide compositions were frequently not binned, leading to biases in the sequence characteristics and gene content of MAGs. They also discovered that genes located in the non-binned regions were strongly biased towards ribosomal RNAs, transfer RNAs, mobile element functions, and genes of unknown function. Conversely, coding sequences with known functions are more likely to be binned. While it is unclear if this preference reflects differences in specific genes, it is plausible to speculate that microorganisms containing particular genes (e.g., the *ureABC* gene) are more likely to be binned. Although this bias may exist during binning, it has a greater impact on the annotation and abundance analyses of non-coding sequences and unknown genes in the microbial communities, and a lesser impact on the analyses of the distribution of known genes in different taxa. Therefore, combining gene abundance and functional annotation of MAGs to jointly determine the abundance of functional genes in microbial communities and their distribution across different taxa is a more reliable method.

High abundance of the *nirB* gene was observed in the lakes, along with high abundance of the *nasA* gene (Figure 1), indicating a high overall abundance of the nitrate reduction pathways. However, genes related to nitrite reduction were more abundant than those related to nitrate reduction. The abundance of nitrogen related genes in the environment is influenced by various environmental factors. Under low oxygen conditions, nitrite concentrations are high and nitrate concentrations are low, leading to increased activity and abundance of nitrite reductase (Canfield et al., 2010). Salinity may also affect the abundance of nitrogen related genes in the environment. For example, the abundance of *nasA* genes was significantly negatively correlated with environmental salinity (Liu et al., 2022). Thus, the relative abundance of nitrate reduction related genes and nitrite reduction related genes varies across different environments. In some settings, nitrate reduction related genes are more abundant, while in others, nitrite reduction related genes are more abundant (Nelson et al., 2015; Ren et al., 2018; Zhang and Lv, 2021; Mosley et al., 2022).

No nitrate-reducing activity has been observed in the nine species of the genus *Yoonia* that have been isolated and cultured, according to the List of Prokaryotic names with Standing in Nomenclature (LPSN)^1^ (Parte et al., 2020). However, *Yoonia* was initially isolated from *Loktanella* in 2018 (Wirth and Whitman, 2018). Notably, nitrate-reducing activity has been observed in species of the genus *Loktanella* that are taxonomically related to *Yoonia* (Lee, 2012). The genus *Yoonia* has been poorly studied, and its nitrite-reducing or nitrogen-fixing enzyme activities are unknown. *Yoonia* has a distinctive distribution, being more abundant in water bodies that are less disturbed by human activities, cold, at high altitudes, or at high latitudes (Zeng et al., 2021, 2022; Feng and Xing, 2023). This unique distribution makes it worthy of further in-depth study. All strains of *Cyclobacterium* obtained from our samples were unknown species. However, previous isolation and culture studies have shown that *C. plantarum* within this genus has positive nitrate and nitrite reduction, with gas formation from nitrate (Shahinpei et al., 2020). Nevertheless, nitrate reduction, but not nitrite reduction, was observed in several species, including *C. amurskyense*; *C. qasimii*; *C. marinum*; *C. lianum*, and *C. caenipelagi* (Jung et al., 2013). Therefore, it was possible that the MAGs we obtained had nitrite-reducing activities but not nitrate-reducing activities, and further experimental verification is needed.

In conclusion, we revealed the structure and the nitrogen cycle of microbial communities in four high-altitude pristine saline lakes in the Altun mountain on the QTP. We observed that *Proteobacteria*, *Bacteroidota*, and *Actinobacteriota* were dominant in these lakes. We recovered 1,601 MAGs, of which 1,440 belong to unknown species, and 1,060 contained nitrogen cycle related genes. This significantly expanded the microbial genomic data resources. We found that the nitrite reduction, nitrogen fixation, and assimilatory nitrate reduction processes may be active in the lakes. Denitrification may be a major mechanism driving the potential nitrogen loss. The dominance of the taxon *Yoonia* in the lake could be attributed to its well-established nitrogen functions. This study contributes to our understanding of microbial diversity and nitrogen function in high-altitude saline lakes on the QTP.

## Data availability statement

The original contributions presented in the study are publicly available. The data can be found here: https://www.ncbi.nlm.nih.gov/bioproject/PRJNA1121972/.

## Author contributions

ZZ: Data curation, Formal analysis, Writing – original draft. YZ: Funding acquisition, Conceptualization, Methodology, Writing – review & editing. FM: Visualization, Writing – review & editing. MX: Investigation, Writing – review & editing. HL: Investigation, Resources, Writing – review & editing. JX: Investigation, Resources, Writing – review & editing. BH: Conceptualization, Supervision, Writing – review & editing. MW: Funding acquisition, Project administration, Supervision, Writing – review & editing.

## Footnotes

^1^ https://lpsn.dsmz.de/

## References

[ref1] Alneberg J. Bjarnason B. S. de Bruijn I. Schirmer M. Quick J. Ijaz U. Z.. (2014). Binning metagenomic contigs by coverage and composition. Nat. Methods 11, 1144–1146. doi: 10.1038/nmeth.3103 25218180

[ref2] Bentzon-Tilia M. Severin I. Hansen Lars H. Riemann L. (2015). Genomics and ecophysiology of heterotrophic nitrogen-fixing Bacteria isolated from estuarine surface water. MBio:6. doi: 10.1128/mbio.00929-15 PMC4495170 26152586

[ref3] Bolger A. M. Lohse M. Usadel B. (2014). Trimmomatic: a flexible trimmer for Illumina sequence data. Bioinformatics 30, 2114–2120. doi: 10.1093/bioinformatics/btu170 24695404 PMC4103590

[ref4] Bottomley P. J. Taylor A. E. Myrold D. D. (2012). A consideration of the relative contributions of different microbial subpopulations to the soil N cycle. Front. Microbiol. 3:373. doi: 10.3389/fmicb.2012.00373 23109931 PMC3478590

[ref5] Bowers R. M. Kyrpides N. C. Stepanauskas R. Harmon-Smith M. Doud D. Reddy T. B. K.. (2017). Minimum information about a single amplified genome (MISAG) and a metagenome-assembled genome (MIMAG) of bacteria and archaea. Nat. Biotechnol. 35, 725–731. doi: 10.1038/nbt.3893 28787424 PMC6436528

[ref6] Brahney J. Mahowald N. Ward D. S. Ballantyne A. P. Neff J. C. (2015). Is atmospheric phosphorus pollution altering global alpine Lake stoichiometry? Global Biogeochem. Cycles 29, 1369–1383. doi: 10.1002/2015gb005137

[ref7] Brunet R. Garcia-Gil L. (1996). Sulfide-induced dissimilatory nitrate reduction to ammonia in anaerobic freshwater sediments. FEMS Microbiol. Ecol. 21, 131–138. doi: 10.1111/j.1574-6941.1996.tb00340.x

[ref8] Canfield D. E. Glazer A. N. Falkowski P. G. (2010). The evolution and future of Earth’s nitrogen cycle. Science 330, 192–196. doi: 10.1126/science.1186120 20929768

[ref9] Capella-Gutierrez S. Silla-Martinez J. M. Gabaldon T. (2009). trimAl: a tool for automated alignment trimming in large-scale phylogenetic analyses. Bioinformatics 25, 1972–1973. doi: 10.1093/bioinformatics/btp348 19505945 PMC2712344

[ref10] Caranto J. D. Lancaster K. M. (2017). Nitric oxide is an obligate bacterial nitrification intermediate produced by hydroxylamine oxidoreductase. Proc. Natl. Acad. Sci. USA 114, 8217–8222. doi: 10.1073/pnas.1704504114 28716929 PMC5547625

[ref11] Casciotti K. L. (2016). Nitrogen and oxygen isotopic studies of the marine nitrogen cycle. Annu. Rev. Mar. Sci. 8, 379–407. doi: 10.1146/annurev-marine-010213-135052 26747521

[ref12] Chaumeil P. A. Mussig A. J. Hugenholtz P. Parks D. H. (2022). GTDB-Tk v2: memory friendly classification with the genome taxonomy database. Bioinformatics 38, 5315–5316. doi: 10.1093/bioinformatics/btac672 36218463 PMC9710552

[ref13] Chen X. Wei H. Zhang J. E. (2021). Nitrogen and sulfur additions improved the diversity of *nirK*- and *nirS*-type denitrifying bacterial communities of farmland soil. Biology (Basel) 10:1191. doi: 10.3390/biology10111191 34827184 PMC8615190

[ref14] Church M. J. Short C. M. Jenkins B. D. Karl D. M. Zehr J. P. (2005). Temporal patterns of nitrogenase gene (*nifH*) expression in the oligotrophic North Pacific Ocean. Appl. Environ. Microbiol. 71, 5362–5370. doi: 10.1128/AEM.71.9.5362-5370.2005 16151126 PMC1214674

[ref15] Daims H. Lebedeva E. V. Pjevac P. Han P. Herbold C. Albertsen M.. (2015). Complete nitrification by *Nitrospira* bacteria. Nature 528, 504–509. doi: 10.1038/nature16461 26610024 PMC5152751

[ref16] Danecek P. Bonfield J. K. Liddle J. Marshall J. Ohan V. Pollard M. O.. (2021). Twelve years of SAMtools and BCFtools. Gigascience 10:giab008. doi: 10.1093/gigascience/giab008 33590861 PMC7931819

[ref17] Díez B. Bergman B. Pedrós-Alió C. Antó M. Snoeijs P. (2012). High cyanobacterial *nifH* gene diversity in Arctic seawater and sea ice brine. Environ. Microbiol. Rep. 4, 360–366. doi: 10.1111/j.1758-2229.2012.00343.x 23760800

[ref18] Ding W. Wang S. Qin P. Fan S. Su X. Cai P.. (2023). Anaerobic thiosulfate oxidation by the *Roseobacter* group is prevalent in marine biofilms. Nat. Commun. 14:2033. doi: 10.1038/s41467-023-37759-4 37041201 PMC10090131

[ref19] Eddy S. R. (2011). Accelerated profile HMM searches. PLoS Comput. Biol. 7:e1002195. doi: 10.1371/journal.pcbi.1002195 22039361 PMC3197634

[ref20] Edgar R. C. (2004). MUSCLE: a multiple sequence alignment method with reduced time and space complexity. BMC Bioinformatics 5, 1–19. doi: 10.1186/1471-2105-5-113 15318951 PMC517706

[ref21] Ettwig K. F. Butler M. K. Le Paslier D. Pelletier E. Mangenot S. Kuypers M. M.. (2010). Nitrite-driven anaerobic methane oxidation by oxygenic bacteria. Nature 464, 543–548. doi: 10.1038/nature08883 20336137

[ref22] Falkowski P. G. (1997). Evolution of the nitrogen cycle and its influence on the biological sequestration of CO_2_ in the ocean. Nature 387, 272–275. doi: 10.1038/387272a0

[ref23] Feng X. Xing P. (2023). Genomics of *Yoonia* sp. isolates (family *Roseobacteraceae*) from Lake Zhangnai on the Tibetan plateau. Microorganisms 11:2817. doi: 10.3390/microorganisms11112817 38004828 PMC10673129

[ref24] Galloway J. N. Aber J. D. Erisman J. W. Seitzinger S. P. Howarth R. W. Cowling E. B.. (2003). The nitrogen cascade. Bioscience 53, 341–356. doi: 10.1641/0006-3568(2003)053[0341:tnc]2.0.co;2

[ref25] Graf D. R. Jones C. M. Hallin S. (2014). Intergenomic comparisons highlight modularity of the denitrification pathway and underpin the importance of community structure for N_2_O emissions. PLoS One 9:e114118. doi: 10.1371/journal.pone.0114118 25436772 PMC4250227

[ref26] Griffin B. M. Schott J. Schink B. (2007). Nitrite, an electron donor for anoxygenic photosynthesis. Science 316:1870. doi: 10.1126/science.1139478 17600210

[ref27] Gruber N. (2008). The marine nitrogen cycle: overview and challenges. Nitrogen Mar. Environ. 2, 1–50.

[ref28] Gruber N. Galloway J. N. (2008). An earth-system perspective of the global nitrogen cycle. Nature 451, 293–296. doi: 10.1038/nature06592 18202647

[ref29] Gurevich A. Saveliev V. Vyahhi N. Tesler G. (2013). QUAST: quality assessment tool for genome assemblies. Bioinformatics 29, 1072–1075. doi: 10.1093/bioinformatics/btt086 23422339 PMC3624806

[ref30] Helen D. Kim H. Tytgat B. Anne W. (2016). Highly diverse *nirK* genes comprise two major clades that harbour ammonium-producing denitrifiers. BMC Genomics 17:155. doi: 10.1186/s12864-016-2465-0 26923558 PMC4770552

[ref31] Heng L. (2013). Aligning sequence reads, clone sequences and assembly contigs with BWA-MEM: arXiv preprint.

[ref32] Huang Y. Fan Z. Zhao C. Chen G. Huang J. Zhou Z.. (2023). Evaluating the impacts of biochemical processes on nitrogen dynamics in a tide gate-controlled river flowing into the South China Sea. Sci. Total Environ. 881:163363. doi: 10.1016/j.scitotenv.2023.163363 37044343

[ref33] Hutchins D. A. Capone D. C. (2022). The marine nitrogen cycle: new developments and global change. Nat. Rev. Microbiol. 20, 401–414. doi: 10.1038/s41579-022-00687-z 35132241

[ref34] Hyatt D. Chen G. L. LoCascio P. F. Land M. L. Larimer F. W. Hauser L. J. (2010). Prodigal: prokaryotic gene recognition and translation initiation site identification. BMC Bioinformatics 11:119. doi: 10.1186/1471-2105-11-119 20211023 PMC2848648

[ref35] Inomura K. Bragg J. Follows M. J. (2017). A quantitative analysis of the direct and indirect costs of nitrogen fixation: a model based on *Azotobacter vinelandii*. ISME J. 11, 166–175. doi: 10.1038/ismej.2016.97 27740611 PMC5315487

[ref36] Jain C. Rodriguez-R L. M. Phillippy A. M. Konstantinidis K. T. Aluru S. (2018). High throughput ANI analysis of 90K prokaryotic genomes reveals clear species boundaries. Nat. Commun. 9:5114. doi: 10.1038/s41467-018-07641-9 30504855 PMC6269478

[ref37] Jiang H. Dong H. Yu B. Lv G. Deng S. Berzins N.. (2009). Diversity and abundance of Ammonia-oxidizing Archaea and Bacteria in Qinghai Lake, northwestern China. Geomicrobiol J. 26, 199–211. doi: 10.1080/01490450902744004

[ref38] Jung Y. T. Lee J. S. Yoon J. H. (2013). *Cyclobacterium caenipelagi* sp. nov., isolated from a tidal flat sediment, and emended description of the genus *Cyclobacterium*. Int. J. Syst. Evol. Microbiol. 63, 3158–3163. doi: 10.1099/ijs.0.050161-0 23435247

[ref39] Jungblut A. D. Neilan B. A. (2010). NifH gene diversity and expression in a microbial mat community on the McMurdo ice shelf, Antarctica. Antarct. Sci. 22, 117–122. doi: 10.1017/S0954102009990514

[ref40] Kanehisa M. Goto S. (2000). KEGG: Kyoto encyclopedia of genes and genomes. Nucleic Acids Res. 28, 27–30. doi: 10.1093/nar/28.1.27 10592173 PMC102409

[ref41] Kang D. W. D. Li F. Kirton E. Thomas A. Egan R. An H.. (2019). MetaBAT 2: an adaptive binning algorithm for robust and efficient genome reconstruction from metagenome assemblies. PeerJ 7:e7359. doi: 10.7717/peerj.7359 31388474 PMC6662567

[ref42] Kartal B. Maalcke W. J. De Almeida N. M. Cirpus I. Gloerich J. Geerts W.. (2011). Molecular mechanism of anaerobic ammonium oxidation. Nature 479, 127–130. doi: 10.1038/nature10453 21964329

[ref43] Katoh K. Standley D. M. (2013). MAFFT multiple sequence alignment software version 7: improvements in performance and usability. Mol. Biol. Evol. 30, 772–780. doi: 10.1093/molbev/mst010 23329690 PMC3603318

[ref44] Kong H. Lin J. Zhang Y. Li C. Xu C. Shen L.. (2023). High natural nitric oxide emissions from lakes on Tibetan plateau under rapid warming. Nat. Geosci. 16, 474–477. doi: 10.1038/s41561-023-01200-8

[ref45] Konneke M. Bernhard A. E. de la Torre J. R. Walker C. B. Waterbury J. B. Stahl D. A. (2005). Isolation of an autotrophic ammonia-oxidizing marine archaeon. Nature 437, 543–546. doi: 10.1038/nature03911 16177789

[ref46] Kuang X. Jiao J. J. (2016). Review on climate change on the Tibetan plateau during the last half century. J. Geophys. Res. Atmos. 121, 3979–4007. doi: 10.1002/2015JD024728

[ref47] Kuypers M. M. M. Marchant H. K. Kartal B. (2018). The microbial nitrogen-cycling network. Nat. Rev. Microbiol. 16, 263–276. doi: 10.1038/nrmicro.2018.9 29398704

[ref48] Lane M. C. Lloyd A. L. Markyvech T. A. Hagan E. C. Mobley H. L. T. (2006). Uropathogenic *Escherichia coli* strains generally lack functional Trg and tap chemoreceptors found in the majority of *E. coli* strains strictly residing in the gut. J. Bacteriol. 188, 5618–5625. doi: 10.1128/jb.00449-06 16855252 PMC1540019

[ref49] Lee S. D. (2012). *Loktanella tamlensis* sp. nov., isolated from seawater. Int. J. Syst. Evol. Microbiol. 62, 586–590. doi: 10.1099/ijs.0.029462-0 21515703

[ref50] Letunic I. Bork P. (2007). Interactive tree of life (iTOL): an online tool for phylogenetic tree display and annotation. Bioinformatics 23, 127–128. doi: 10.1093/bioinformatics/btl529 17050570

[ref51] Li D. H. Liu C. M. Luo R. B. Sadakane K. Lam T. W. (2015). MEGAHIT: an ultra-fast single-node solution for large and complex metagenomics assembly via succinct de Bruijn graph. Bioinformatics 31, 1674–1676. doi: 10.1093/bioinformatics/btv033 25609793

[ref52] Li B. Wang L. Li H. Xue J. Luo W. Xing P.. (2024). Phosphorus-driven regime shift from heterotrophic to autotrophic diazotrophs in a deep alpine lake. Water Res. 248:120848. doi: 10.1016/j.watres.2023.120848 37976949

[ref53] Lin G. Lin X. (2022). Bait input altered microbial community structure and increased greenhouse gases production in coastal wetland sediment. Water Res. 218:118520. doi: 10.1016/j.watres.2022.118520 35525032

[ref54] Liu Q. Yang J. Wang B. Liu W. Hua Z. Jiang H. (2022). Influence of salinity on the diversity and composition of carbohydrate metabolism, nitrogen and sulfur cycling genes in lake surface sediments. Front. Microbiol. 13:1019010. doi: 10.3389/fmicb.2022.1019010 36519167 PMC9742235

[ref55] Lockwood C. Butt J. N. Clarke T. A. Richardson D. J. (2011). Molecular interactions between multihaem cytochromes: probing the protein-protein interactions between pentahaem cytochromes of a nitrite reductase complex. Biochem. Soc. Trans. 39, 263–268. doi: 10.1042/bst0390263 21265785

[ref56] Lu S.-J. Si J.-H. Hou C.-Y. Li Y.-S. Wang M.-M. Yan X.-X.. (2017). Spatiotemporal distribution of nitrogen and phosphorus in alpine lakes in the Sanjiangyuan region of the Tibetan plateau. Water Sci. Technol. 76, 396–412. doi: 10.2166/wst.2017.091 28726705

[ref57] Macdonald H. Pope N. R. Cole J. A. (1985). Isolation, characterization and complementation analysis of *nirB* mutants of *Escherichia Coli* deficient only in NADH-dependent nitrite reductase activity. J. Gen. Microbiol. 131, 2771–2782. doi: 10.1099/00221287-131-10-2771 3906030

[ref58] Malm S. Tiffert Y. Micklinghoff J. Schultze S. Joost I. Weber I.. (2009). The roles of the nitrate reductase NarGHJI, the nitrite reductase NirBD and the response regulator GlnR in nitrate assimilation of *Mycobacterium tuberculosis*. Microbiology (Reading) 155, 1332–1339. doi: 10.1099/mic.0.023275-0 19332834

[ref59] Martínez-Espinosa R. M. Cole J. A. Richardson D. J. Watmough N. J. (2011). Enzymology and ecology of the nitrogen cycle. Biochem. Soc. Trans. 39, 175–178. doi: 10.1042/BST0390175 21265768

[ref60] Milanese A. Mende D. R. Paoli L. Salazar G. Ruscheweyh H. J. Cuenca M.. (2019). Microbial abundance, activity and population genomic profiling with mOTUs2. Nat. Commun. 10:1014. doi: 10.1038/s41467-019-08844-4 30833550 PMC6399450

[ref61] Moriya Y. Itoh M. Okuda S. Yoshizawa A. C. Kanehisa M. (2007). KAAS: an automatic genome annotation and pathway reconstruction server. Nucleic Acids Res. 35, W182–W185. doi: 10.1093/nar/gkm321 17526522 PMC1933193

[ref62] Mortazavi A. Williams B. A. McCue K. Schaeffer L. Wold B. (2008). Mapping and quantifying mammalian transcriptomes by RNA-Seq. Nat. Methods 5, 621–628. doi: 10.1038/nmeth.1226 18516045 PMC13303166

[ref63] Mosley O. E. Gios E. Close M. Weaver L. Daughney C. Handley K. M. (2022). Nitrogen cycling and microbial cooperation in the terrestrial subsurface. ISME J. 16, 2561–2573. doi: 10.1038/s41396-022-01300-0 35941171 PMC9562985

[ref64] Nelson M. B. Berlemont R. Martiny A. C. Martiny J. B. H. (2015). Nitrogen cycling potential of a grassland litter microbial community. Appl. Environ. Microbiol. 81, 7012–7022. doi: 10.1128/AEM.02222-15 26231641 PMC4579426

[ref65] Nelson M. B. Martiny A. C. Martiny J. B. (2016). Global biogeography of microbial nitrogen-cycling traits in soil. Proc. Natl. Acad. Sci. USA 113, 8033–8040. doi: 10.1073/pnas.1601070113 27432978 PMC4961168

[ref66] Nelson W. C. Tully B. J. Mobberley J. M. (2020). Biases in genome reconstruction from metagenomic data. PeerJ 8:e10119. doi: 10.7717/peerj.10119 33194386 PMC7605220

[ref67] Pachiadaki M. G. Sintes E. Bergauer K. Brown J. M. Record N. R. Swan B. K.. (2017). Major role of nitrite-oxidizing bacteria in dark ocean carbon fixation. Science 358, 1046–1051. doi: 10.1126/science.aan8260 29170234

[ref68] Pajares S. Ramos R. (2019). Processes and microorganisms involved in the marine nitrogen cycle: knowledge and gaps. Front. Mar. Sci. 6:739. doi: 10.3389/fmars.2019.00739

[ref69] Parks D. H. Imelfort M. Skennerton C. T. Hugenholtz P. Tyson G. W. (2015). CheckM: assessing the quality of microbial genomes recovered from isolates, single cells, and metagenomes. Genome Res. 25, 1043–1055. doi: 10.1101/gr.186072.114 25977477 PMC4484387

[ref70] Parte A. C. Carbasse J. S. Meier-Kolthoff J. P. Reimer L. C. Goker M. (2020). List of prokaryotic names with standing in nomenclature (LPSN) moves to the DSMZ. Int. J. Syst. Evol. Microbiol. 70, 5607–5612. doi: 10.1099/ijsem.0.004332 32701423 PMC7723251

[ref71] Qiu J. (2008). China: the third pole. Nature 454, 393–396. doi: 10.1038/454393a 18650887

[ref72] R Core Team. (2013). R: a language and environment for statistical computing.

[ref73] Ren M. Zhang Z. Wang X. Zhou Z. Chen D. Zeng H.. (2018). Diversity and contributions to nitrogen cycling and carbon fixation of soil salinity shaped microbial communities in tarim basin. Front. Microbiol. 9:431. doi: 10.3389/fmicb.2018.00431 29593680 PMC5855357

[ref74] Richter M. Rossello-Mora R. (2009). Shifting the genomic gold standard for the prokaryotic species definition. Proc. Natl. Acad. Sci. USA 106, 19126–19131. doi: 10.1073/pnas.0906412106 19855009 PMC2776425

[ref75] Shahinpei A. Amoozegar M. A. Mirfeizi L. Nikou M. M. Ventosa A. Sánchez-Porro C. (2020). Taxogenomics of the genus *Cyclobacterium*: *Cyclobacterium xiamenense* and *Cyclobacterium halophilum* as synonyms and description of *Cyclobacterium plantarum* sp. nov. Microorganisms 8:610. doi: 10.3390/microorganisms8040610 32340290 PMC7232363

[ref76] Shen W. Le S. Li Y. Hu F. Q. (2016). SeqKit: a cross-platform and ultrafast toolkit for FASTA/Q file manipulation. PLoS One 11:e0163962. doi: 10.1371/journal.pone.0163962 27706213 PMC5051824

[ref77] Sieber C. M. K. Probst A. J. Sharrar A. Thomas B. C. Hess M. Tringe S. G.. (2018). Recovery of genomes from metagenomes via a dereplication, aggregation and scoring strategy. Nat. Microbiol. 3, 836–843. doi: 10.1038/s41564-018-0171-1 29807988 PMC6786971

[ref78] Simon J. Klotz M. G. (2013). Diversity and evolution of bioenergetic systems involved in microbial nitrogen compound transformations. Biochim. Biophys. Acta 1827, 114–135. doi: 10.1016/j.bbabio.2012.07.005 22842521

[ref79] Simon J. van Spanning R. J. M. Richardson D. J. (2008). The organisation of proton motive and non-proton motive redox loops in prokaryotic respiratory systems. Biochim. Biophys. Acta 1777, 1480–1490. doi: 10.1016/j.bbabio.2008.09.008 18930017

[ref80] Sohm J. A. Webb E. A. Capone D. G. (2011). Emerging patterns of marine nitrogen fixation. Nat. Rev. Microbiol. 9, 499–508. doi: 10.1038/nrmicro2594 21677685

[ref81] Stamatakis A. (2014). RAxML version 8: a tool for phylogenetic analysis and post-analysis of large phylogenies. Bioinformatics 30, 1312–1313. doi: 10.1093/bioinformatics/btu033 24451623 PMC3998144

[ref82] Thompson A. W. Foster R. A. Krupke A. Carter B. J. Musat N. Vaulot D.. (2012). Unicellular cyanobacterium symbiotic with a single-celled eukaryotic alga. Science 337, 1546–1550. doi: 10.1126/science.1222700 22997339

[ref83] Tu Q. He Z. Wu L. Xue K. Xie G. Chain P.. (2017). Metagenomic reconstruction of nitrogen cycling pathways in a CO_2_-enriched grassland ecosystem. Soil Biol. Biochem. 106, 99–108. doi: 10.1016/j.soilbio.2016.12.017

[ref84] Vaksmaa A. Guerrero-Cruz S. van Alen T. A. Cremers G. Ettwig K. F. Lüke C.. (2017). Enrichment of anaerobic nitrate-dependent methanotrophic “*Candidatus* Methanoperedens nitroreducens” archaea from an Italian paddy field soil. Appl. Microbiol. Biotechnol. 101, 7075–7084. doi: 10.1007/s00253-017-8416-0 28779290 PMC5569662

[ref85] Van Kessel M. A. Speth D. R. Albertsen M. Nielsen P. H. Op den Camp H. J. Kartal B.. (2015). Complete nitrification by a single microorganism. Nature 528, 555–559. doi: 10.1038/nature16459 26610025 PMC4878690

[ref86] Van Trappen S. Mergaert J. Swings J. (2004). *Loktanella salsilacus* gen. Nov., sp. nov., *Loktanella fryxellensis* sp. nov and *Loktanella vestfoldensis* sp. nov., new members of the *Rhodobacter* group, isolated from microbial mats in Antarctic lakes. Int. J. Syst. Evol. Microbiol. 54, 1263–1269. doi: 10.1099/ijs.0.03006-0 15280301

[ref87] Villanueva R. A. M. Chen Z. J. (2019). ggplot2: elegant graphics for data analysis. *Meas.: Interdiscip*. Res. Pers. 17, 160–167. doi: 10.1080/15366367.2019.1565254

[ref88] Vreca P. Muri G. (2006). Changes in accumulation of organic matter and stable carbon and nitrogen isotopes in sediments of two Slovenian mountain lakes (Lake Ledvica and Lake Planina), induced by eutrophication changes. Limnol. Oceanogr. 51, 781–790. doi: 10.4319/lo.2006.51.1_part_2.0781

[ref89] Wang H. N. Gunsalus R. P. (2000). The nrfA and nirB nitrite reductase operons in *Escherichia coli* are expressed differently in response to nitrate than to nitrite. J. Bacteriol. 182, 5813–5822. doi: 10.1128/jb.182.20.5813-5822.2000 11004182 PMC94705

[ref90] Wang M. Wu J. Zhou T. Liang Y. Zheng L. X. Sun Y. X. (2021). Effects of copper and florfenicol on *nirS*- and *nirK*-type denitrifier communities and related antibiotic resistance in vegetable soils. Ecotoxicol. Environ. Saf. 213:112011. doi: 10.1016/j.ecoenv.2021.112011 33592374

[ref91] Wang L. Xing P. Li H. Zhou L. Wu Q. L. (2020). Distinct intra-lake heterogeneity of diazotrophs in a deep oligotrophic mountain lake. Microb. Ecol. 79, 840–852. doi: 10.1007/s00248-019-01461-0 31811330

[ref92] Wei C. Sun D. Yuan W. L. Li L. Dai C. X. Chen Z. Z.. (2023). Metagenomics revealing molecular profiles of microbial community structure and metabolic capacity in Bamucuo lake, Tibet. Environ. Res. 217:114847. doi: 10.1016/j.envres.2022.114847 36402183

[ref93] Wirth J. S. Whitman W. B. (2018). Phylogenomic analyses of a clade within the roseobacter group suggest taxonomic reassignments of species of the genera *Aestuariivita*, *Citreicella*, *Loktanella*, *Nautella*, *Pelagibaca*, *Ruegeria*, *Thalassobius*, *Thiobacimonas* and *Tropicibacter*, and the proposal of six novel genera. Int. J. Syst. Evol. Microbiol. 68, 2393–2411. doi: 10.1099/ijsem.0.002833 29809121

[ref94] Wolfe A. P. Baron J. S. Cornett R. J. (2001). Anthropogenic nitrogen deposition induces rapid ecological changes in alpine lakes of the Colorado front range (USA). J. Paleolimnol. 25, 1–7. doi: 10.1023/a:1008129509322

[ref95] Wu Y. Wang S. Ni Z. Li H. May L. Pu J. (2021). Emerging water pollution in the world’s least disturbed lakes on Qinghai-Tibetan plateau. Environ. Pollut. 272:116032. doi: 10.1016/j.envpol.2020.116032 33218770

[ref96] Wunsch P. Zumft W. G. (2005). Functional domains of NosR, a novel transmembrane iron-sulfur flavoprotein necessary for nitrous oxide respiration. J. Bacteriol. 187, 1992–2001. doi: 10.1128/jb.187.6.1992-2001.2005 15743947 PMC1064061

[ref97] Yang J. Jiang H. Dong H. Hou W. Li G. Wu G. (2015). Sedimentary archaeal *amoA* gene abundance reflects historic nutrient level and salinity fluctuations in Qinghai Lake, Tibetan plateau. Sci. Rep. 5:18071. doi: 10.1038/srep18071 26666501 PMC4678299

[ref98] Yang J. Jiang H. Dong H. Wang H. Wu G. Hou W.. (2013). *amoA*-encoding archaea and thaumarchaeol in the lakes on the northeastern Qinghai-Tibetan plateau, China. Front. Microbiol. 4:329. doi: 10.3389/fmicb.2013.00329 24273535 PMC3824093

[ref99] Yang J. Jiang H. Wu G. Hou W. Sun Y. Lai Z.. (2012). Co-occurrence of nitrite-dependent anaerobic methane oxidizing and anaerobic ammonia oxidizing bacteria in two Qinghai-Tibetan saline lakes. Front. Earth Sci. 6, 383–391. doi: 10.1007/s11707-012-0336-9

[ref100] Zehr J. P. Mellon M. T. Zani S. (1998). New nitrogen-fixing microorganisms detected in oligotrophic oceans by amplification of Nitrogenase (*nifH*) genes. Appl. Environ. Microbiol. 64, 3444–3450. doi: 10.1128/AEM.64.9.3444-3450.1998 9726895 PMC106745

[ref101] Zeng Y. X. Li H. R. Luo W. (2022). Gene transfer agent *g5* gene reveals bipolar and endemic distribution of *Roseobacter* clade members in polar coastal seawater. Diversity (Basel) 14:392. doi: 10.3390/d14050392

[ref102] Zeng Y. X. Luo W. Li H. R. Yu Y. (2021). High diversity of planktonic prokaryotes in Arctic Kongsfjorden seawaters in summer 2015. Polar Biol. 44, 195–208. doi: 10.1007/s00300-020-02791-3

[ref103] Zhang X. Lin C. Zhou X. Lei K. Guo B. Cao Y.. (2019). Concentrations, fluxes, and potential sources of nitrogen and phosphorus species in atmospheric wet deposition of the Lake Qinghai watershed, China. Sci. Total Environ. 682, 523–531. doi: 10.1016/j.scitotenv.2019.05.224 31129540

[ref104] Zhang L. Lv J. (2021). Land-use change from cropland to plantations affects the abundance of nitrogen cycle-related microorganisms and genes in the loess plateau of China. Appl. Soil Ecol. 161:103873. doi: 10.1016/j.apsoil.2020.103873

[ref105] Zhang S. Qin W. Bai Y. Zhang Z. Wang J. Gao H.. (2021). Linkages between anammox and denitrifying bacterial communities and nitrogen loss rates in high-elevation rivers. Limnol. Oceanogr. 66, 765–778. doi: 10.1002/lno.11641

[ref106] Zhang X. Yao C. Zhang B. Tan W. Gong J. Wang G.-Y.. (2023). Dynamics of benthic nitrate reduction pathways and associated microbial communities responding to the development of seasonal deoxygenation in a coastal mariculture zone. Environ. Sci. Technol. 57, 15014–15025. doi: 10.1021/acs.est.3c03994 37756318

[ref107] Zhao Y. Liu Z. Zhang B. Cai J. Yao X. Zhang M.. (2023). Inter-bacterial mutualism promoted by public goods in a system characterized by deterministic temperature variation. Nat. Commun. 14:5394. doi: 10.1038/s41467-023-41224-7 37669961 PMC10480208

