# Supplementary material for: The microbial community structure and nitrogen cycle of high-altitude pristine saline lakes on the Qinghai-Tibetan plateau
Source: Front Microbiol. 2024 Jul 9;15:1424368. doi: 10.3389/fmicb.2024.1424368 (PMC11312105; doi:10.3389/fmicb.2024.1424368)
Supplement: Supplementary file 1 [file Data_Sheet_1.docx]

**Supplementary Figures 1-12**

**Supplementary Figure 1.** Locations of the four high-altitude pristine saline lakes in the Altun mountain on the Qinghai-Tibetan Plateau. Source: https://www.arcgis.com/apps/instant/atlas/index.html.

**Supplementary Figure 2.** Locations of the sampling sites of Lake Aqqikkol. Source: https://www.arcgis.com/apps/instant/atlas/index.html.

**Supplementary Figure 3.** Locations of the sampling sites of Lake Ayakkum. Source: https://www.arcgis.com/apps/instant/atlas/index.html.

**Supplementary Figure 4.** Locations of the sampling sites of Lake Jingyu. Source: https://www.arcgis.com/apps/instant/atlas/index.html.

**Supplementary Figure 5.** Locations of the sampling sites of Lake Wusuxiao. Source: https://www.arcgis.com/apps/instant/atlas/index.html.


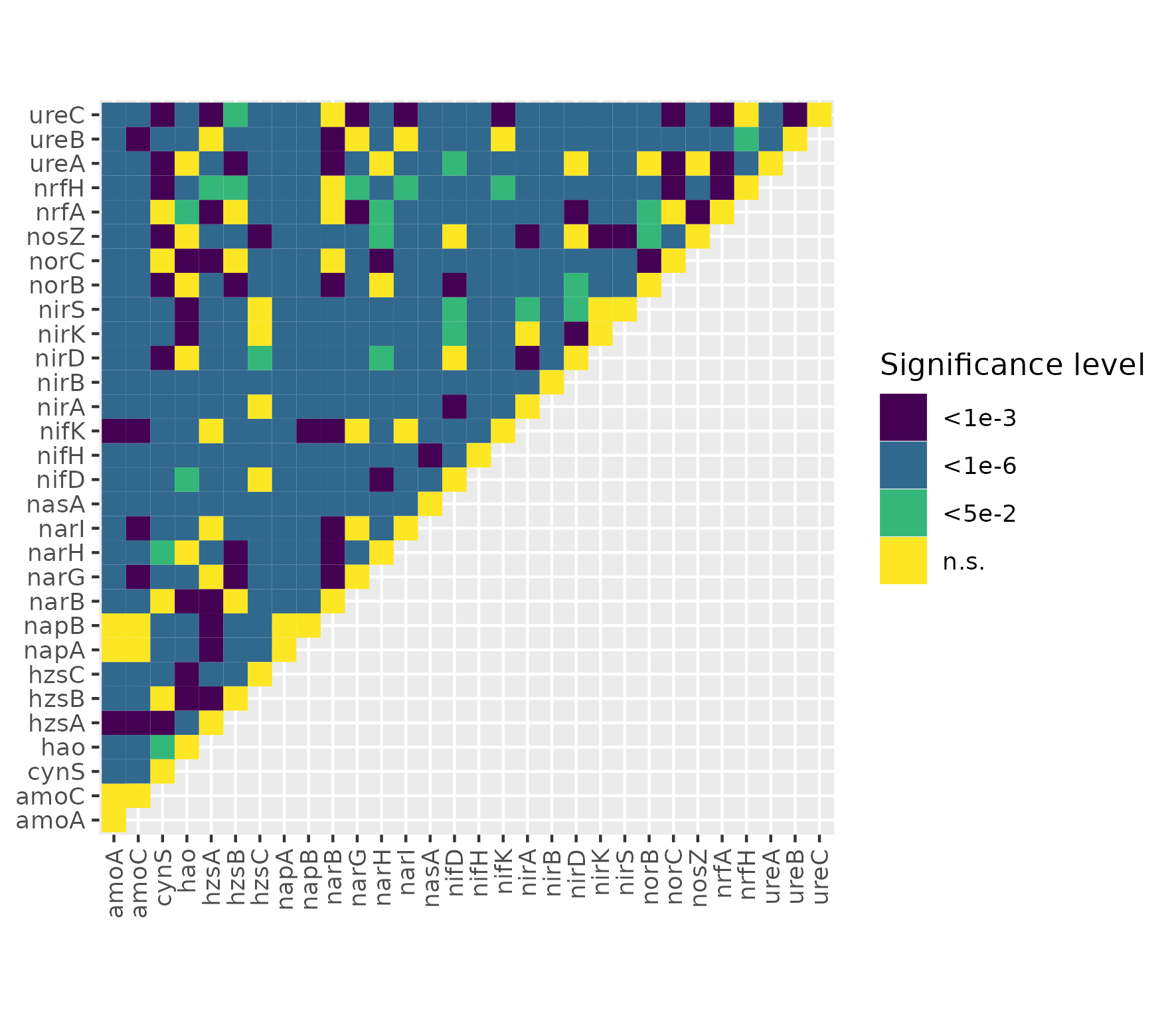


**Supplementary Figure 6.** P-value of the comparison among the depths of nitrogen cycle related genes.

**Supplementary Figure 7.** Abundance of nitrogen cycle related genes in each lake. (A) Lake Aqqikkol. (B) Lake Ayakkum. (C) Lake Jingyu. (D) Lake Wusuxiao.


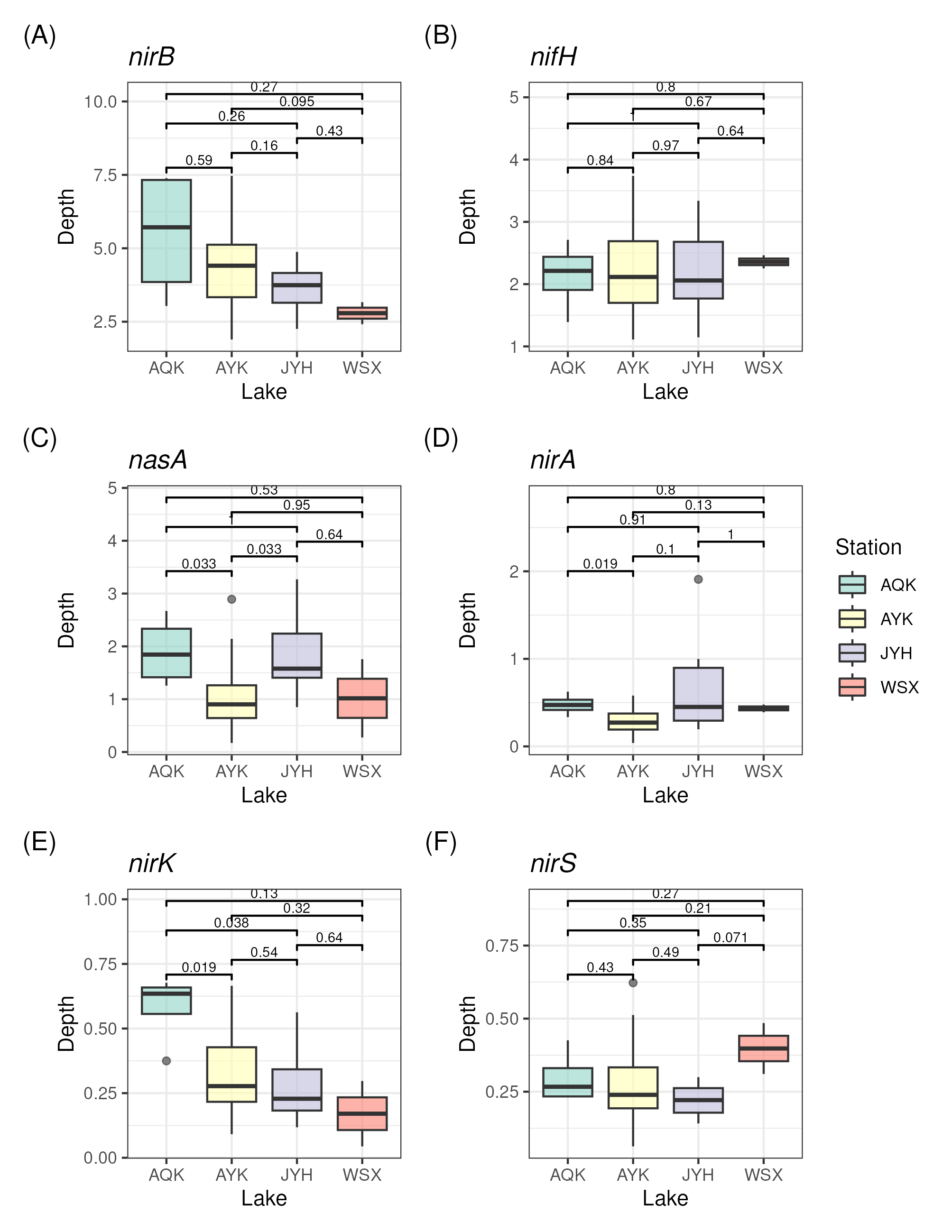


**Supplementary Figure 8.** Abundance of the six most abundant genes in each lake. (A) the *nirB* gene. (B) the *nifH* gene. (C) the *nasA* gene. (D) the *nirA* gene. (E) the *nirK* gene. (F) the *nirS* gene.


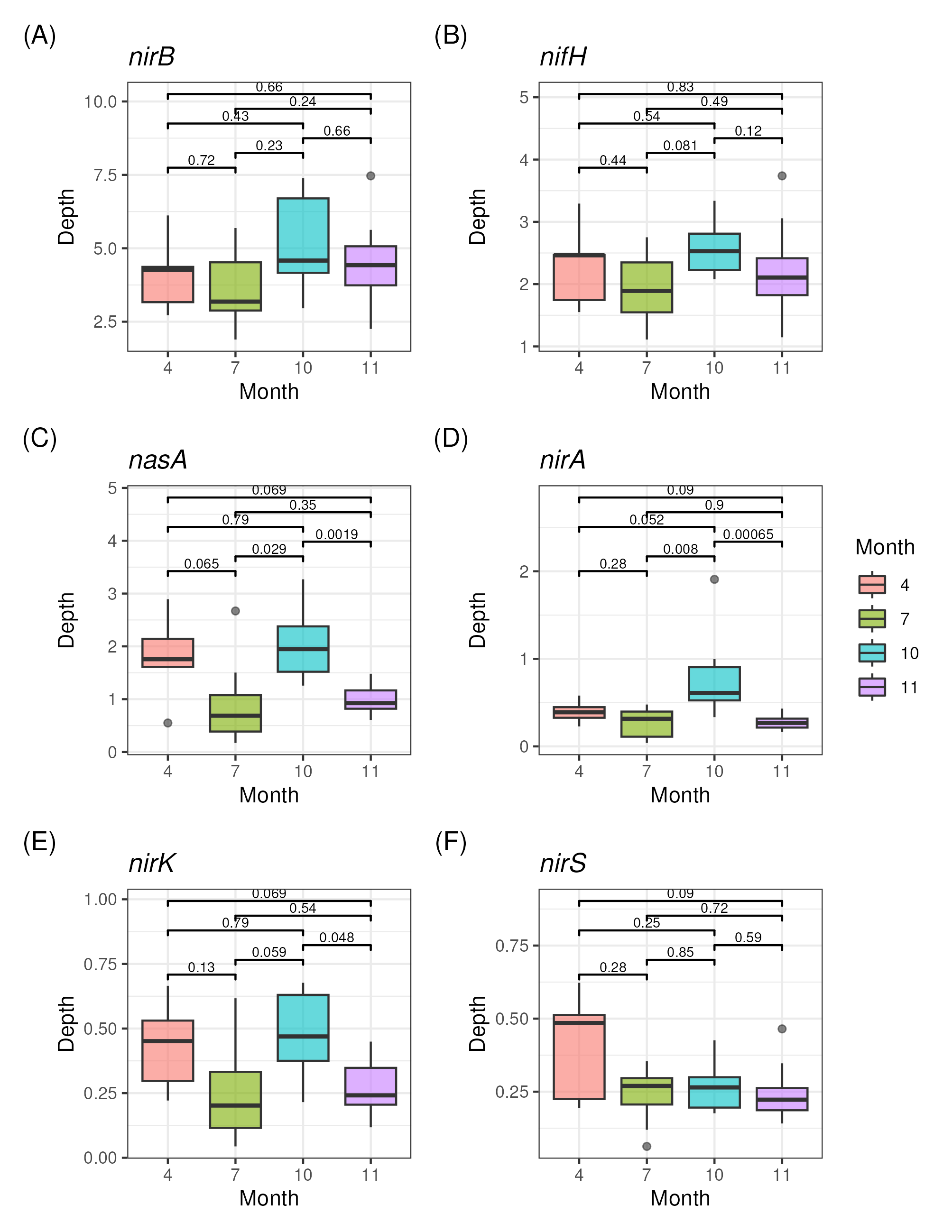


**Supplementary Figure 9.** Abundance of the six most abundant genes in each sampling month. (A) the *nirB* gene. (B) the *nifH* gene. (C) the *nasA* gene. (D) the *nirA* gene. (E) the *nirK* gene. (F) the *nirS* gene.


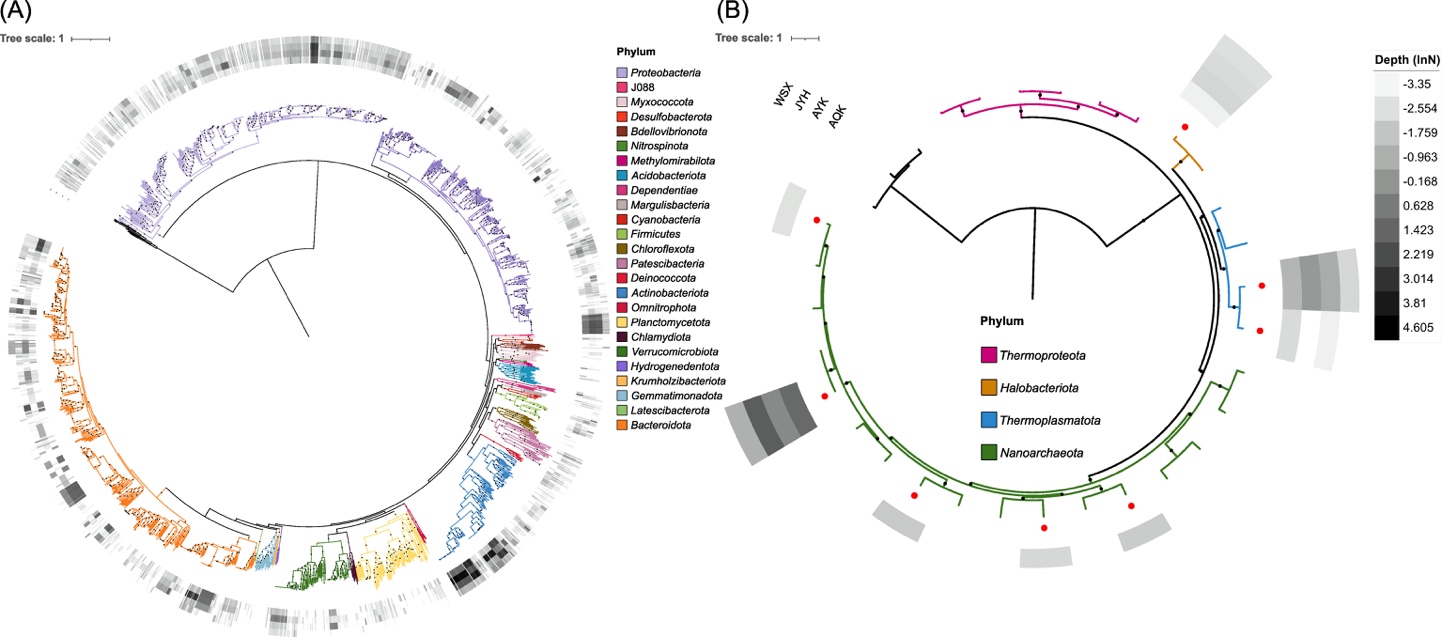


**Supplementary Figure 10.** Phylogenetic trees of metagenome-assembled genomes (MAGs). (A) The phylogenetic tree of bacterial MAGs. (B) The phylogenetic tree of archaeal MAGs. Red dots show MAGs. The colors of the branches show different phyla. Solid circles on the branches represent bootstrap values ≥ 70% from 100 replicates. The outer circle shows the abundance of the MAG in each lake. From the center to the outside: Lake Aqqikkol, Lake Ayakkum, Lake Jingyu, Lake Wusuxiao.

**Supplementary Figure 11.** *Yoonia’*s contribution in nitrogen cycle related genes in each sample. (A) the *nifH* gene. (B) the *nir*K gene. (C) the *nasA* gene.


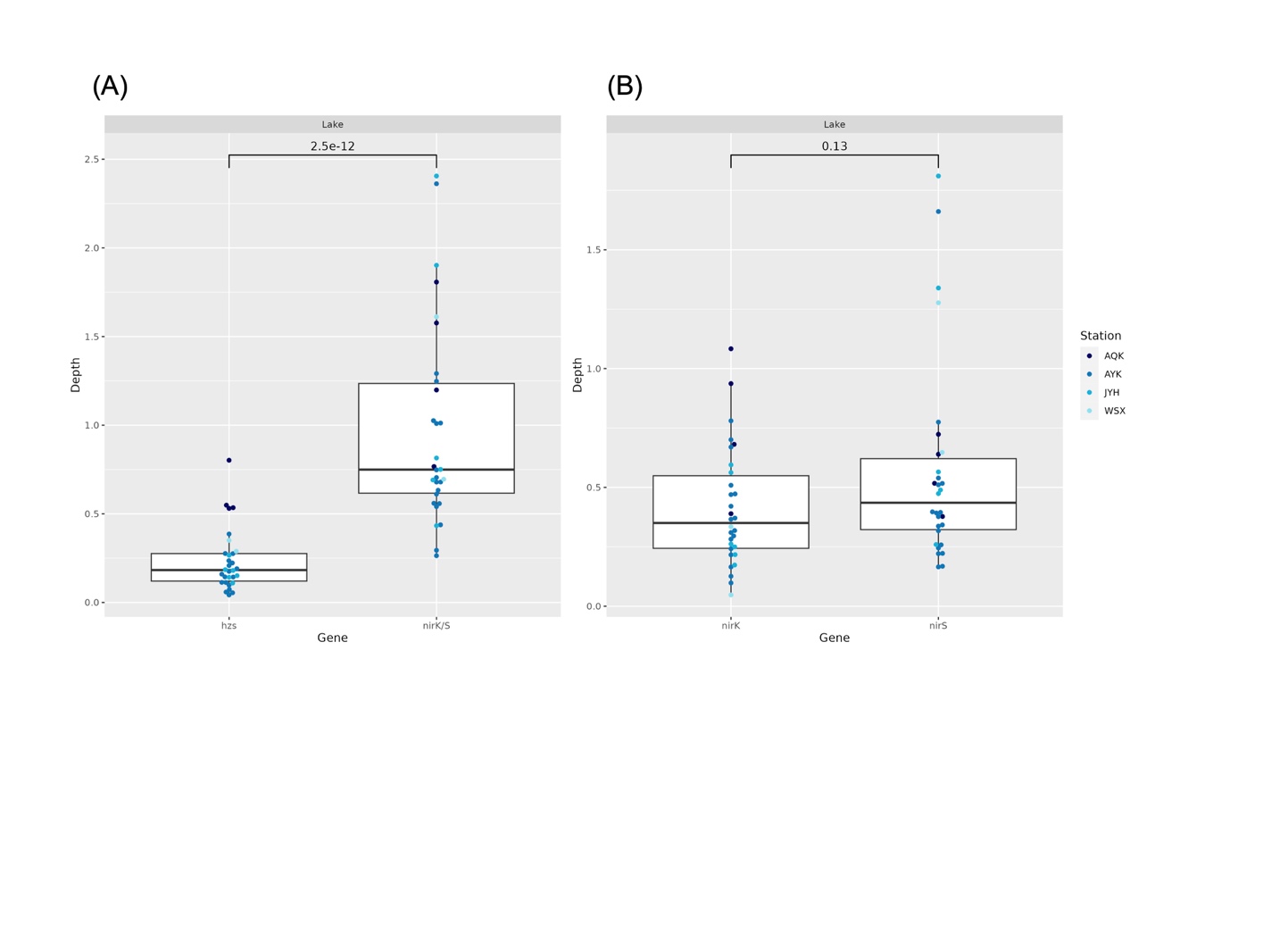


**Supplementary Figure 12.** Comparison of the depth of genes associated with nitrogen loss in the high-altitude pristine saline lakes. (a) The hydrazine synthase gene (*hzs*) and the nitrite reductase genes (*nirK*/*nirS*). (b) The *nirK* gene and *nirS* gene.
